# PSSMHCpan: a novel PSSM-based software for predicting class I peptide-HLA binding affinity

**DOI:** 10.1093/gigascience/gix017

**Published:** 2017-03-15

**Authors:** Geng Liu, Dongli Li, Zhang Li, Si Qiu, Wenhui Li, Cheng-chi Chao, Naibo Yang, Handong Li, Zhen Cheng, Xin Song, Le Cheng, Xiuqing Zhang, Jian Wang, Huanming Yang, Kun Ma, Yong Hou, Bo Li

**Affiliations:** 1 BGI Education Center, University of Chinese Academy of Sciences, Main Building, Beishan Industrial Zone, Yantian District, Shenzhen 518083, China; 2 BGI-Shenzhen, Main Building, Beishan Industrial Zone, Yantian District, Shenzhen 518083, China; 3 BGI-GenoImmune, Gaoxing road, East Lake New Technology Development Zone, Wuhan 430079, China; 4 Complete Genomics, Inc., 2071 Stierlin Court, Mountain View, CA 94043, USA; 5 Molecular Imaging Program at Stanford, Department of Radiology and Bio-X Program, Stanford University, Montag Hall, 355 Galvez Street, Stanford, CA 94305, USA; 6 The Third Affiliated Hospital of Kunming Medical University (Tumor Hospital of Yunnan Province), Kunzhou Road, Xishan District, Kunming 650100, Yunnan Province, China; 7 BGI-Yunnan, Haiyuan North Road, Kunming Hi-tech Development Zone, Kunming 650000, Yunnan Province, China; 8 James D. Watson Institute of Genome Sciences, Yuhang Tong Road, Xihu District, Hangzhou 310058, Zhejiang Province, China; 9 Department of Biology, University of Copenhagen, Nørregade 10, PO Box 2177, 1017 Copenhagen K, Denmark; 10 BGI-Forensics, Main Building, Beishan Industrial, Zone Yantian District, Shenzhen 518083, China

**Keywords:** Antitumor vaccine, peptide-HLA binding affinity, PSSMHCpan, neoantigen

## Abstract

Predicting peptide binding affinity with human leukocyte antigen (HLA) is a crucial step in developing powerful antitumor vaccine for cancer immunotherapy. Currently available methods work quite well in predicting peptide binding affinity with HLA alleles such as HLA-A*0201, HLA-A*0101, and HLA-B*0702 in terms of sensitivity and specificity. However, quite a few types of HLA alleles that are present in the majority of human populations including HLA-A*0202, HLA-A*0203, HLA-A*6802, HLA-B*5101, HLA-B*5301, HLA-B*5401, and HLA-B*5701 still cannot be predicted with satisfactory accuracy using currently available methods. Furthermore, currently the most popularly used methods for predicting peptide binding affinity are inefficient in identifying neoantigens from a large quantity of whole genome and transcriptome sequencing data. Here we present a Position Specific Scoring Matrix (PSSM)-based software called PSSMHCpan to accurately and efficiently predict peptide binding affinity with a broad coverage of HLA class I alleles. We evaluated the performance of PSSMHCpan by analyzing 10-fold cross-validation on a training database containing 87 HLA alleles and obtained an average area under receiver operating characteristic curve (AUC) of 0.94 and accuracy (ACC) of 0.85. In an independent dataset (Peptide Database of Cancer Immunity) evaluation, PSSMHCpan is substantially better than the popularly used NetMHC-4.0, NetMHCpan-3.0, PickPocket, Nebula, and SMM with a sensitivity of 0.90, as compared to 0.74, 0.81, 0.77, 0.24, and 0.79. In addition, PSSMHCpan is more than 197 times faster than NetMHC-4.0, NetMHCpan-3.0, PickPocket, sNebula, and SMM when predicting neoantigens from 661 263 peptides from a breast tumor sample. Finally, we built a neoantigen prediction pipeline and identified 117 017 neoantigens from 467 cancer samples of various cancers from TCGA. PSSMHCpan is superior to the currently available methods in predicting peptide binding affinity with a broad coverage of HLA class I alleles.

## Introduction

Cancer immunotherapy has been proven in recent years to be a promising strategy that enhances the strengths of the immune system of cancer patients to fight cancer. This strategy exploits the fact that the surface of cancer cells has a variety of tumor antigens (i.e., peptides of 8–13 residues in length) coming from various kinds of mutated proteins cleaved by the proteasomes intracellular. These peptides are bound to HLA class I allelic specific molecules, forming peptide-HLA complexes which are presented to T cell receptors (TCRs). If TCRs can recognize these peptide-HLA complexes on the surface of cancer cells, cytotoxic T lymphocytes will destroy cancer cells. Cancer cells are highly heterogeneous in terms of morphological, phonotypical, and genetic profiles. Cancer cells of different tumors and within the same tumor could present hundreds of different types of peptides. The immune system of cancer patients could only recognize small populations of cancer cells. To enhance the power of the cytotoxic T lymphocytes to recognize and eradicate as many cancer cells as possible, one strategy is to vaccinate cancer patients with complex antitumor peptides. The first step to develop powerful antitumor vaccines is to predict peptide binding affinity with HLA class I allele.

To predict peptide binding affinity with HLA class I alleles, four types of methods have been developed, including structure-based methods, machine learning-based methods, PSSM-based methods [16], and combined methods. The structure-based methods predict peptide binding affinity calculating the minimum free energy of peptide-HLA complex [30], which allows us to understand the peptide-HLA binding affinity at the structure level. However, the predicting speed of this type of methods is extremely slow and inaccurate due to limited number of available crystal structures [20]. The machine learning-based methods predict peptide binding affinity by learning a function that maps a given peptide to areas with binding affinity based on available known bound peptides (binders). Because machine learning-based methods can accurately predict peptides with specific HLA alleles such as HLA-A*0201, HLA-A*0101, and HLA-B*0702 [25, 41], they are frequently used in many studies [8, 37, 40]. Thus far, many machine learning-based methods have been developed, including support vector machine-based method MHC2PRED [15], hidden markov model-based method S-HMM [26], artificial neural network-based method NetMHC [2, 17], and pan-specific method NetMHCpan [11, 23, 24]. Although currently available tools can predict a number of HLA class I allelic coverage with appreciable area under receiver operating characteristic curve (AUC), they cannot predict with satisfactory accuracy quite a few types of HLA alleles that are present in the majority of human populations. For example, NetMHC, ARB, Nebula, sNebula, and SMM achieved only the average predicted AUC of no more than 0.85 when they were used in predicting HLA-A*0202, HLA-A*0203, HLA-A*6802, HLA-B*5101, HLA-B*5301, HLA-B*5401, and HLA-B*5701 [19, 21, 27]. Further, these methods are inefficient in predicting a large quantity of peptides generated from whole genome and transcriptome sequencing data because of their nonlinear computation complexity. In contrast, PSSM-based methods predict peptide binding affinity by building a matrix from a multiple peptide alignment that represents the motif information (i.e., the binding anchor). These methods can predict binding affinity fast, because PSSM’s linear computational complexity is much less complex than the nonlinear computational complexity of structure-based and machine learning-based methods. Based on the mechanism of PSSM, several software packages have been developed such as PickPocket [42], SVMHC [9], and nHLAPred [5]. However, the predicting accuracy of this software is not as good as that of machine learning-based methods [42]. Recently, to predict peptide-HLA binding affinity more accurately, scientists from several groups combined different methods to develop new software, including NetMHCcons [13], IEDB [34], and HLaffy [22]. Although these combined methods indeed have shown a better predictive performance as compared to individual methods, their predictive accuracy is still not satisfactory, especially in clinical applications [4]. To develop more effective immunotherapy, it is necessary to develop better software that can more accurately and efficiently predict peptide binding affinity with a broad coverage of HLA class I alleles.

Here, we present a novel software called PSSMHCpan that can predict peptide binding affinity accurately and efficiently. We designed this software based on the PSSM mechanism and trained it with a larger database containing 63 519 peptide-HLA pairs which allow us to allele specifically predict peptide binding affinity with HLA class I allele. To predict peptide binding affinity with a broad coverage of HLA class I alleles, we induce a simple but powerful pan-specific prediction approach based on the similarity of HLA protein sequences. We show that PSSMHCpan can accurately and efficiently predict peptide binding affinity with a broad HLA class I allelic coverage of at least 87 types in 10-fold cross-validation, and it performed better than the other 5 software packages when evaluated with Peptide Database of Cancer Immunity dataset. Finally, we built a prediction pipeline to identify neoantigens in 467 TCGA tumor samples across 10 types of cancers.

## Methods

PSSM is represented as a motif of multiple sequence alignment result [39]. The basic principle of PSSMHCpan is that peptides that bind to a specific HLA allele possess the motif information that can be studied by PSSM. We propose the PSSMHCpan in two novel aspects. Firstly, we construct a comprehensive training database and build allele-specific PSSMs for predicting peptide binding affinity with a characterized HLA class I allele (with binders in training database). Secondly, we use the similarity of HLA sequences to induce a simple but powerful pan-specific prediction approach based on our hypothesis below, and predict peptide binding affinity with uncharacterized HLA class I allele (without binders in training database).

**Figure 1:**
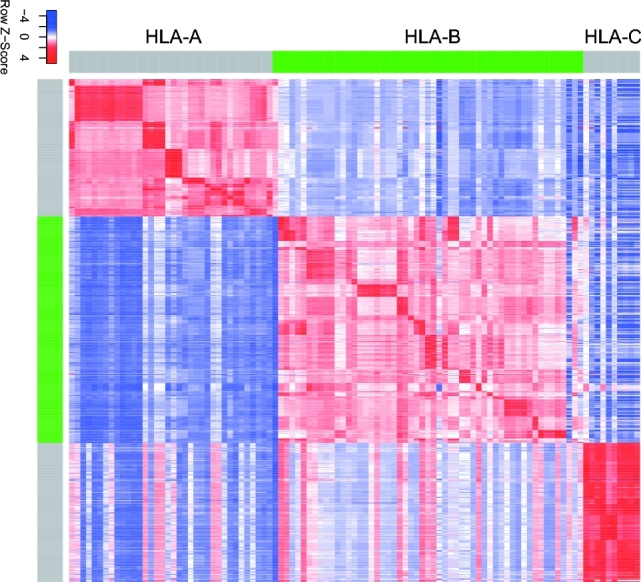
Heat map of HLA protein sequence similarity. The larger the Z-Score, the more similar of the pair HLA protein sequences. It showed high similarity between different types of HLA alleles within the same gene locus.

**Figure 2:**
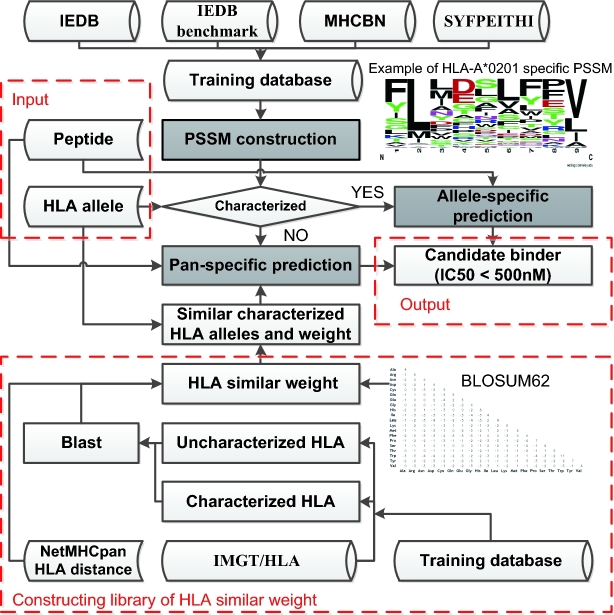
Method of PSSMHCpan. The three main steps are shown in grey background.

It is well known that peptides on the cell surface are bound to the floor of the peptide-binding groove that is in the central region of the α1/α2 heterodimer (a molecule composed of two nonidentical subunits) of HLA protein sequences [33]. By analyzing the sequences of HLA proteins, we noticed that HLA protein sequences are highly similar among different HLA alleles (Fig. 1), suggesting that peptides bound to similar HLA alleles have similar binding affinity according to the predictive value of IC50. Thereby, we hypothesize that since different HLA protein sequences are similar, the peptide binding affinity with different HLA alleles should be similar too. Based on this hypothesis and the PSSM mechanism, we designed the software PSSMHCpan in the following three steps: PSSM construction, allele-specific prediction, and pan-specific prediction. The flowchart of PSSMHCpan is shown in Fig. 2.

### PSSM construction

We define PSSM as a matrix of M rows (amino acid; M = 20) and N columns (length; N = 8 ∼ 25). Each element *P_ai_*in the matrix is the likelihood of a given character (amino acid) at its position. We calculate the element *P_ai_* through the following function,

\[
{P_{ai}} = \log \frac{{{F_{ai}} + \omega }}{{B{G_a}}},
\]

where *F_ai_* denotes the frequency of amino acid *a* at position *i* from the training database; *BG_a_* denotes the background frequency of amino acid *a* from UniProt database [3]; and ω is a random value (ranging from 0 to 1) generated from Dirichlet distribution [1].

### Allele-specific prediction

To qualitatively predict peptide binding affinity with characterized HLA allele, we define a *binding_score* as the sum of the corresponding values of each amino acid of a given peptide at each position in the corresponding allele-specific PSSM.

\[
{\rm{binding}}\_{\rm{score}} = \frac{{\mathop \sum \nolimits_{{\rm{i}} = 1}^{\rm{N}} {{\rm{P}}_{{\rm{ai}}}}}}{N}
\]

We consider a peptide with *binding_score* > 0 as a binder according to the signal prediction of GeneID [10]. The higher *binding_score* that a peptide has, the higher binding affinity this peptide would have.

We converted a *binding_score* into an IC50 value as follows:

\[
{\rm {IC}50} = 50000 \, {^{\rm {Max \, - \, {{binding}{\_score}}}}}/{\rm {Max - Min}},
\]

where Max and Min denote the maximum and the minimum values of *binding_score*, respectively. In this study, we assigned Max as 0.8 and Min as −0.8 based on our experience. According to the recommendation of IEDB [43], we consider a peptide with IC50 < 500 nM as a binder and a peptide with IC50 < 50 nM as a strong binder.

### Pan-specific prediction

Firstly, we construct a library of HLA similar weight (button panel in Fig. 2) that contains pairs of characterized and uncharacterized HLA alleles, and each pair has a weight value. We determine a pair of characterized and uncharacterized HLA alleles by using BLOSUM62-based [32] BLAST alignment of HLA protein sequences and assign the alignment score as the weight value. We also extracted the nearest distance of HLA alleles from NetMHCpan-3.0 [23] as a pair of characterized and uncharacterized HLA alleles and assigned a constant as the weight value.

Secondly, we qualitatively predict the binding affinity of a given peptide with uncharacterized HLA allele with an *IC50_un_* value which is calculated as below:

\[
IC{50_{un}} = \frac{{\mathop \sum \nolimits_{{\rm{i}} = 1}^{\rm{S}} ({{\rm{w}}_{\rm{i}}^{*}}\ {\rm{IC}}{{50}_{\rm{i}}})}}{{\mathop \sum \nolimits_{{\rm{i}} = 1}^{\rm{S}} {{\rm{w}}_{\rm{i}}}}},
\]

where S denotes the sum of characterized HLA alleles that pair up the specific uncharacterized HLA allele according to the library of HLA similar weight. *w_i_* and *IC*50_*i*_ denote the weight value and the allele-specific prediction result of peptide binding affinity with HLA allele *i*. We also consider a peptide with IC50_un_ < 500 nM as a binder, and a peptide with IC50_un_ < 50 nM as a strong binder.

### 10-fold cross-validation

We applied 10-fold cross-validation [4] to evaluate the performance of peptide-HLA binding prediction as follows. Firstly, we randomly partitioned our collected experientially verified binders (see Data description) into 10 subsets of nearly equal size. Subsequently, we performed 10 iterations of training and validation. In each iteration, we use a different subset of data for validation, while the remaining 9 subsets for training. To evaluate specificity, we also added the nearly same number of nonbinders (see Data description) to our subset of data for validation. In another word, each validation dataset consist of nearly equal number of binders and nonbinders.

### Data description

We collected our training database of HLA class I binders from the following resources: the Immune Epitope Database and Analysis Resource (IEDB) [36], IEDB benchmark [14], SYFPEITHI [31], MHCBN [6], and in-house experimental epitopes. After filtering out duplications and peptides with abnormal amino acids which do not or rarely exist naturally, such as B, J, O, U, X, and Z, we obtained 64 677 peptide-HLA pairs that cover 162 HLA alleles (Table 1). We selected only HLA alleles that consist of at least 10 binders with a fixed length. Finally, we built 241 PSSMs for allele-specific prediction of peptides with variable lengths (8 ∼ 25 peptides) bound to 123 HLA class I alleles (Additional file 1: Table S1).

**Table 1:** Summary of training database.

Database	IEDB	IEDB benchmark	SYFPEITHI	MHCBN	Combined	Training database
HLA alleles	166	95	109	103	162	123
Binders	54 272	40 930	3329	4070	64 677	63 519

We selected 60 530 binders covering 87 HLA class I alleles from our training database for 10-fold cross-validation. To evaluate specificity, we collected 60 102 nonbinders that include experimentally verified ones from IEDB benchmark [14] and computer randomly constructed ones predicted as nonbinders by any of the following four methods: PSSMHCpan, NetMHC-4.0, NetMHCpan-3.0, and PickPocket. We use computer-constructed nonbinders because currently available experimentally verified nonbinders that meet our requirement only cover 50 HLA class I alleles.

We collected 64 uncharacterized HLA class I alleles that cannot be predicted with NetMHC-4.0 but can be predicted with NetMHCpan-3.0. We extracted 2064 binders that bind to the 64 uncharacterized HLA alleles from our training database and 2057 nonbinders as a Dataset for Pan-specific evaluation (DP).

To construct a library of HLA weight similarity, we collected 690 497 pairs of characterized and uncharacterized HLA class I alleles from 13 957 HLA protein sequences in IMGT/HLA (Release 3.23.0) [29], and 2800 pairs from the nearest distance of HLA alleles in NetMHCpan-3.0, respectively. After removing duplications, we retained 691 031 pairs for pan-specific prediction of peptide binding affinity with 4896 HLA class I alleles (Additional file 1: Table S1).

We also collected an independent dataset of binders from the Peptide Database of Cancer Immunity [35]. From this database, we selected 285 binders that cover 38 HLA alleles of HLA-A, HLA-B, and HLA-C. After removing duplications, we retained 273 binders for validation.

To detect pan-cancer neoantigens, we obtained somatic mutations from 467 TCGA tumor samples across 10 cancer types (Table 2) from GDC data portal (https://gdc-portal.nci.nih.gov/), and the RSEM gene expression data in these tumors and in their paired normal tissues from FireBrowse (http://firebrowse.org/). In addition, we also obtained the RNASeq aligned bam files from these tumors from dbGAP.

**Table 2:** Summary of 467 cancer samples from TCGA cohort.

Cancer type	Patient no.	Cancer type	Patient no.
BLCA	19	LIHC	47
BRCA	93	LUAD	57
COAD	16	PRAD	43
HNSC	39	STAD	28
KIRC	67	THCA	58

**Figure 3:**
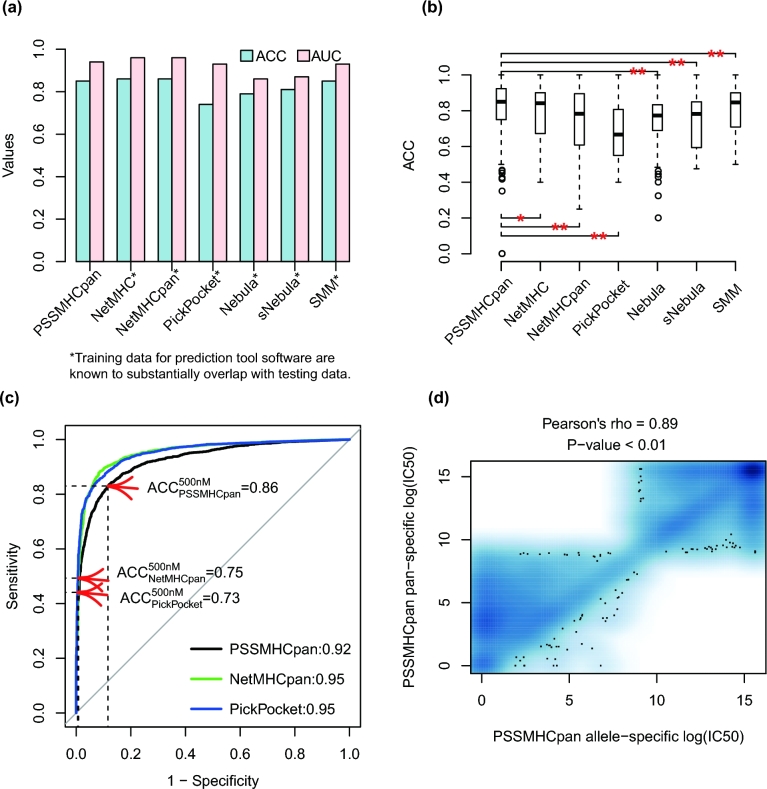
Evaluation on broad HLA allelic coverage. (**a**) The allele-specific prediction evaluation results showed AUC and ACC values of PSSMHCpan, and also compared to NetMHC-4.0, NetMHCpan-3.0, PickPocket, Nebula, sNebula, and SMM. (**b**) The boxplot of individual ACC of a particular HLA allele with fixed peptide length. Comparison between PSSMHCpan and the other six methods was performed by using paired *t* test. “*” denotes *P* < 0.05 and “**” denotes *P* < 0.01. (**c**) The evaluation results showed by ROC of PSSMHCpan in pan-specific prediction, NetMHCpan-3.0, and PickPocket. The ACC, sensitivity, and specificity at cutoff of 500 nM were also shown. (**d**) Correlation analysis of peptide-HLA binding affinity result of IC50 value in log2 between allele-specific prediction and pan-specific prediction.

### Analyses

#### Evaluation of peptide binding affinity prediction with a broad HLA class I allelic coverage

To evaluate the allele-specific prediction accuracy of PSSMHCpan with a broad HLA class I allelic coverage, we performed 10-fold cross-validation on training data of 87 HLA class I alleles that contain at least 12 binders, and obtained an average AUC of 0.94 and prediction accuracy ACC (${\rm{ACC}} = \frac{{{\rm{TP}} + {\rm{TN}}}}{{{\rm{TP}} + {\rm{FP}} + {\rm{TN}} + {\rm{FN}}}}$, where TP, FP, TN, and FN represent true-positive, false-positive, true-negative, and false-negative) of 0.85 under a cutoff of 500 nM. We then used the same validation data to evaluate six popularly used software packages: NetMHC-4.0, NetMHCpan-3.0, PickPocket, Nebula [18], sNebula [19], and SMM [28], respectively. It is worth noting that the training data of these six software packages are from IEDB, IEDB benchmark, MHCBN, SYFPEITHI, and so on [2, 18, 19, 23, 28, 42], which are largely overlapped (>65%) with the validation data in our 10-fold cross-validation analysis. Despite this substantial overlap (which will biasedly increase the AUC values for these software), we found that the AUC values of our PSSMHCpan are slightly lower than those of NetMHC-4.0 and NetMHCpan-3.0, but higher than those of PickPocket, Nebula, sNebula, and SMM (Fig. 3a; Additional file 1: Table S2). By comparing the ACC of each HLA allele with fixed peptide length among the seven software packages, we found that the median ACC of PSSMHCpan is significantly larger than other software (*P* < 0.05, paired *t* test; Fig. 3b). When looking at the AUC value of specific HLA and peptide length, PSSMHCpan not only achieved the substantially results of at least 0.93 in previous good prediction HLA alleles such as HLA-A*0101, HLA-A*0201, and HLA-B*0702, but also performed well in other HLA alleles such as HLA-A*0202, HLA-A*0203, HLA-A*6802, HLA-B*5301, HLA-B*5401, and HLA-B*5701 (Table 3).

**Table 3:** Assessments (AUC values) of peptide binding affinity prediction with specific HLA alleles and peptide length by PSSMHCpan, NetMHC, NetMHCpan, PickPocket, Nebula, sNebula, and SMM.

HLA	Length	PSSMHCpan	NetMHC*	NetMHCpan*	PickPocket*	Nebula*	sNebula*	SMM*
A*0101	9	0.96	0.98	0.98	0.94	0.82	0.97	0.97
A*0101	10	0.94	0.98	0.97	0.94	0.69	0.96	0.98
A*0201	9	0.93	0.94	0.94	0.94	0.88	0.93	0.94
A*0201	10	0.96	0.96	0.97	0.96	0.94	0.97	0.96
B*0702	9	0.95	0.97	0.97	0.96	0.81	0.95	0.97
B*0702	10	0.94	0.98	0.97	0.96	0.80	0.93	0.98
A*0202	9	0.96	0.99	0.99	0.97	0.53	0.89	0.98
A*0203	9	0.97	0.98	0.99	0.98	0.85	0.97	0.98
A*0203	10	0.95	0.98	0.98	0.95	0.53	0.96	0.97
A*6802	9	0.93	0.98	0.98	0.95	0.80	0.95	0.97
A*6802	10	0.91	0.96	0.96	0.92	0.78	0.97	0.97
B*5101	10	0.82	0.89	0.90	0.87	0.72	0.96	0.89
B*5301	9	0.93	0.98	0.98	0.96	0.55	0.88	0.98
B*5301	10	0.91	0.97	0.95	0.92	0.69	0.91	0.97
B*5401	9	0.91	0.98	0.97	0.95	0.51	0.89	0.98
B*5401	10	0.87	0.97	0.97	0.96	0.53	0.88	0.99
B*5701	9	0.98	0.99	0.99	0.98	0.94	0.99	0.99

*Training data are substantially overlapped with validation data.

Considering that a one-time 10-fold cross-validation of randomly selection and nonbinders construction might produce biased results, we repeated another five times the 10-fold cross-validation and found that the standard deviations (SD) of AUC are ≤0.0001, indicating no bias in the 10-fold cross-validation (Table 4).

**Table 4:** The AUC and SD values in 5 times 10-fold cross-validation.

Time	1	2	3	4	5	SD
PSSMHCpan	0.9405	0.9405	0.9408	0.9405	0.9406	0.0001

To evaluate pan-specific prediction of PSSMHCpan, we removed peptides in the DP dataset (see Data description) from our training data and retrained PSSMHCpan. We then predicted those peptides with PSSMHCpan, and obtained an AUC of 0.92 and an ACC of 0.86. We also predicted those peptides with NetMHCpan-3.0 and PickPocket, which gave AUC values of 0.95 and ACC values of 0.75 and 0.73, respectively. It is worth noting that the peptides that we predicted with PSSMHCpan, NetMHCpan-3.0, and PickPocket are removed from our training data but included in the training data of NetMHCpan-3.0 and PickPocket.

To evaluate the pan-specificity of PSSMHCpan, we compared the allele-specific prediction with pan-specific prediction of 3408 correctly predicted peptides in DP dataset. We observed a high correlation between allele-specific and pan-specific prediction (Pearson's rho = 0.89, *P* < 0.01; Fig. 3d), suggesting that our PSSMHCpan can quantitatively predict peptide-HLA binding affinity with profound accuracy.

Mukherjee et al. (2016) recently published a peptide binding affinity prediction software HLaffy that was evaluated with peptides from MHCBN and correctly detected 1179 of 1323 binders (Table 5). To compare the performance of our PSSMHCpan with that of HLaffy, we removed the peptides in MHCBN from our training database and retrained our PSSMHCpan with the remaining peptides. Because nonbinders are much less than binders in MHCBN, we only used the binders in MHCBN to evaluate and calculated the prediction accuracy by sensitivity (${\rm{Sen}} = \frac{{TP}}{{{\rm{TP}} + {\rm{FP}}}}$). We found that our PSSMHCpan correctly identified 1309 of 1323 binders (Table 5).

**Table 5:** Comparison of PSSMHCpan with HLaffy. The prediction of HLaffy was performed on webserver (http://proline.biochem.iisc.ernet.in/HLaffy/).

Allele	PSSMHCpan	HLaffy
HLA-A*0201	1.00	0.92
HLA-A*0203	1.00	0.93
HLA-A*0206	1.00	0.93
HLA-A*0301	1.00	0.84
HLA-A*1101	1.00	0.96
HLA-A*2402	1.00	0.77
HLA-A*3301	1.00	0.83
HLA-A*6801	1.00	0.94
HLA-A*6802	0.95	0.73
HLA-B*0702	1.00	0.88
HLA-B*3501	0.99	0.89
HLA-B*5301	1.00	0.92
HLA-B*5401	1.00	0.88
All	0.99	0.90

#### Evaluation of peptide binding affinity prediction with an independent dataset

**Figure 4:**
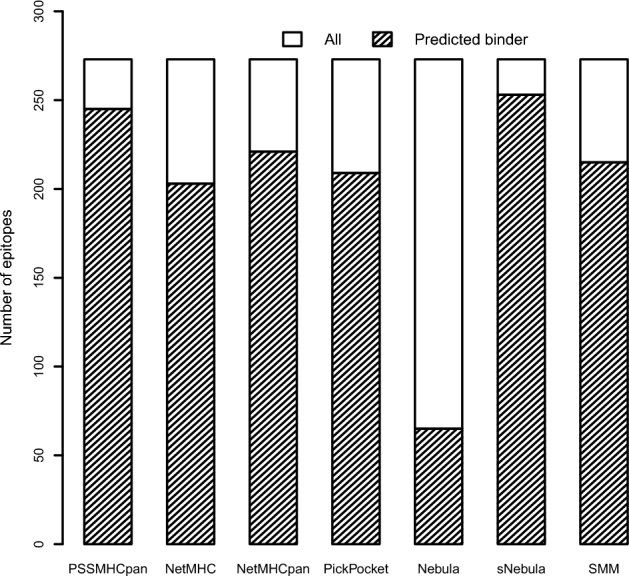
The evaluation result of the independent dataset. We denoted IC50 < 500 nM as binder in PSSMHCpan, NetMHC, NetMHCpan, PickPocket, and SMM. In Nebula prediction, value ≧1.5 as binder. In sNebula prediction, value ≧0 as binder.

Considering cross-validation might overestimate prediction accuracy, we reevaluated PSSMHCpan, NetMHC-4.0, NetMHCpan-3.0, PickPocket, Nebula, sNebula, and SMM with an independent dataset that contains 273 nonduplicated experimentally verified binders from the Peptide Database of Cancer Immunity. Of the 273 binders, 238 are included in our training data. To perform independent evaluation, we firstly removed the 238 binders from our training data, and then retrained the PSSMHCpan with the remaining training data. Together, we identified 268 of 273 (0.98) binders with seven software packages. Of the 268 binders identified, PSSMHCpan and sNebula identified (245 and 253) substantially more binders than the other five software packages did (Fig. 4; Additional file 1: Table S4).

#### Evaluation of the peptide binding affinity prediction efficiency

As whole genome sequencing and whole exome sequencing of cancer genome data are rapidly increasing, there is an urgent need to develop software that can quickly identify neoantigens from cancer genome data. To compare the efficiency of PSSMHCpan, NetMHC-4.0, NetMHCpan-3.0, PickPocket, Nebula, sNebula, and SMM, we first calculated the predicting speed of 10-fold cross-validation on a training database with 87 HLA class I alleles and found that PSSMHCpan is much faster than the other six (ranging from 1.7 to 291.9 times faster; Table 6). We then used each software package to independently predict binding affinity of 661 263 peptides generated from a breast tumor sample that contains 3062 somatic mutations with 6 HLA class I alleles. We found that PSSMHCpan completed the analysis in about 6 seconds. In contrast, NetMHC-4.0 took 3.61 hours, NetMHCpan-3.0 took 28.63 hours, PickPocket took 1.34 hours, sNebula took 0.35 hours, and SMM took 1.49 hours to complete the analysis. Apparently, PSSMHCpan is far more efficient than other methods in detecting neoantigens from a large quantity of sequencing data.

**Table 6:** The predicting speed (CPU time) of the seven software packages. The fastest ones were marked in bold.

	10-fold	Breast tumour
	cross-	neoantigens
Methods	validation	prediction
PSSMHCpan	18.40 s	6.34 s
NetMHC-4.0	1056.83 s	13 001.57 s
NetMHCpan-3.0	5371.16 s	103 060.24 s
PickPocket	282.83 s	4839.63 s
Nebula	146.70 s	Not done
sNebula	31.04 s	1245.88 s
SMM	222.45 s	5369.36 s

CPU time measured in second(s).

#### Pan-cancer neoantigens

**Figure 5:**
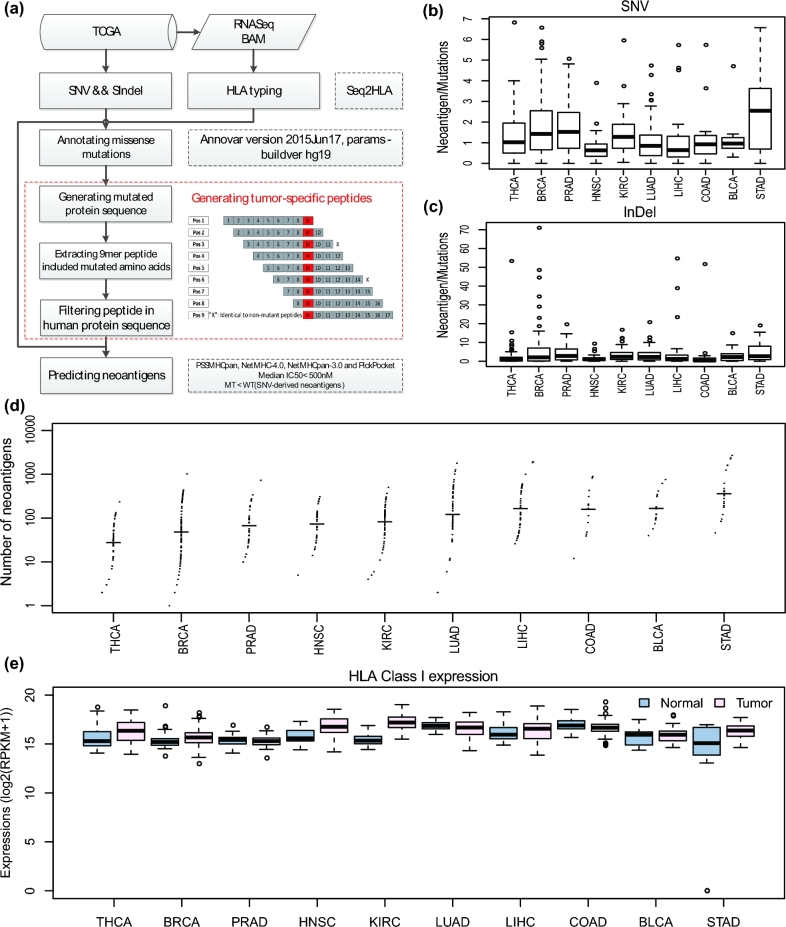
Pan-cancer neoantigens. (**a**) The flow-char of neoantigen prediction pipeline. Software with parameters using in the pipeline are shown in dashed line. (**b**) The distribution of neoantigens generated from each SNV across diverse cancers. (**c**) The distribution of neoantigens generated from each InDel across diverse cancers. (**d**) The distribution of neoantigen loads across 10 cancer types. The cancer types are sorted by median value of neoantigen loads. (**e**) The expression of HLA class I in tumor and corresponding normal samples.

To identify neoantigens that can be used as candidate markers to develop antitumor vaccine, we developed a neoantigen prediction pipeline to determine what types of mutated peptides in cancer cells could be brought to the cell surface by HLAs based on somatic small mutations (SSMs). To maximize prediction accuracy, we included PSSMHCpan, NetMHC-4.0, NetMHCpan-3.0, and PickPocket into our pipeline to detect neoantigens in TCGA tumor samples as follows (Fig. 5a). We first annotated missense SSMs including single nucleotide variants (SNVs) and insertions and deletions (InDels) with ANNOVAR [38] to create a list of tumor-specific peptides (8–13) with an in-house script. After HLA alleles are predicted with Seq2HLA [7], we predict neoantigens with PSSMHCpan, NetMHC-4.0, NetMHCpan-3.0, and PickPocket, respectively. Finally, we selected a list of candidate neoantigens that met the following conditions: (i) predicting as binders (IC50 < 500 nM) by at least two software packages and taking the median value of IC50 as final result; (ii) the IC50 value of a given SNV-derived neoantigen must be smaller than that of its corresponding wild-type peptide [12]. Using this pipeline, we analyzed the neoantigens across 10 cancer types from the TCGA cohort.

In total we identified 117 017 candidate neoantigens from 467 TCGA cancer samples. We calculated the number of candidate neoantigens per SSM in different types of cancer and observed that STAD, PRAD, and BRCA had the highest neoantigens with 2.54, 1.52, and 1.43 per SNV, respectively (Fig. 5b), whereas the highest neoantigens per InDel were 2.76, 2.59, and 2.34 in PRAD, STAD, and KIRC, respectively (Fig. 5c). We also compared the neoantigen loads (number of candidate neoantigens per sample) across 10 cancer types and found that STAD, COAD, and BLCA tumors had the highest neoantigen loads with median values of 302, 182, and 163, while the THCA tumors had a lowest median neoantigen load of 30 (Fig. 5d).

On average we identified 251 candidate neoantigens in each tumor. We then investigated whether the expression level of HLA class I would be increased in cancer cells to bind neoantigens. Indeed, by looking at the mRNA expression in 467 TCGA tumor samples and their paired normal tissues, we found that the expression of HLA class I was markedly elevated in most tumors (Fig. 5e). Since the amount of candidate neoantigens differs substantially among different tumors, we examined whether the number of candidate neoantigens was correlated with HLA class I expression level in each tumor. However, we found no correlation between the number of candidate neoantigens and the HLA class I expression levels in tumors (Pearson's rho = −0.05, *P* = 0.33).

## Discussion

Designing antitumor vaccine requires predicting peptide-HLA binding affinity with high accuracy. In this article, we have presented a novel software, PSSMHCpan, that allows us to predict peptide binding affinity with a broad coverage of HLA class I alleles. By comparing our PSSMHCpan with NetMHC-4.0, NetMHCpan-3.0, PickPocket, Nebula, sNebula, and SMM, we demonstrate that overall our PSSMHCpan is at least as good as the other six in predicting peptide-HLA binding affinity in terms of accuracy, and PSSMHCpan is far more efficient in detecting neoantigens from a large quantity of sequencing data.

In recent years, PSSM-based methods to predict peptide-HLA binding affinity were gradually replaced by machine learning-based methods that are believed to have reliable accuracy and larger data prediction capability [20]. However, by comparing our PSSMHCpan with machine learning-based methods NetMHC-4.0 and NetMHCpan-3.0, we show that our PSSMHCpan exhibits a higher predicting accuracy than NetMHC-4.0 and NetMHCpan-3.0 as evidenced by the independent dataset evaluation. In terms of data prediction capability, PSSMHCpan can allele-specifically and pan-specifically predict peptides that bind to 123 and 4896 HLA class I alleles, respectively, while NetMHC-4.0 and NetMHCpan-3.0 can predict only 89 and 2924 HLA class I alleles, respectively. Furthermore, the PSSMHCpan displays more than 2050 and 16 255 times higher prediction efficiency as compared to NetMHC-4.0 and NetMHCpan-3.0 (Table 6).

Practically, we noticed that the size of the training database appeared to directly affect the prediction accuracy. We believe that a larger training database could have improved the prediction accuracy of PSSMHCpan. For instance, the PSSMHCpan prediction accuracy ACC in predicting 9mer peptides bind to HLA-A*0101 and HLA-B*5703 are 0.96 and 0.70. Not surprisingly, there are 813 binders for HLA-A*0101 and only 25 binders for HLA-B*5703, respectively, in our training data.

It is worth noting that PSSMs with fewer training binders may contain more zero elements (i.e., amino acid “X” was never observed at position “Y”), which is represented as random omega in the formula of “PSSM construction” that could affect the prediction accuracy. We investigated what training binder sizes have less random omega in PSSMs, and how training binder sizes could affect prediction accuracy. There are 6784 9mer peptides bound to HLA-A*0201 in our training database. We randomly selected 678 (10%) binders from the 6784 9mer peptides for predicting. We then repeatedly predicted peptide binding affinity of the same 678 binders with PSSMHCpan respectively trained with increasing sizes of binders with an increment step of 10, randomly selected from the remaining 6106 binders. We found that the prediction accuracy was increased as the training sizes increased, and the prediction accuracy reached a plateau when the sizes of training binders are >100 (Additional file 1: Table S5). This suggests that PSSMHCpan trained with fewer than 100 binders would contain fewer random omegas and have stable prediction accuracy. There are fewer than 100 training binders in 145 of 241 PSSMs in our PSSMHCpan. In our 10-fold cross-validation, PSSMs with fewer than 100 training binders could have increased or decreased AUC, with a mean value of 0.88 (ranging from 0.5 to 1). In the case of the independent dataset evaluation, 3 of 273 binders are incorrectly predicted due to PSSMs with fewer than 100 training binders.

Based on the evaluation results (Fig. 4), we recognized that none of the available software is perfect and that to maximize the peptide binding affinity prediction accuracy, it is necessary to use multiple software packages. We believe that to provide actionable neoantigens that can be used in cancer immunotherapy, it requires more efforts to validate the function and immunogenicity of the predicted neoantigens experimentally.

In conclusion, our PSSMHCpan can predict peptide binding affinity with a broad coverage of HLA class I alleles accurately and far more efficiently compared with the currently most popular peptide binding affinity prediction software. Our PSSMHCpan can not only help develop personalized antitumor vaccines, but also has great potential in other aspects of cancer immunotherapy, including designing dendritic cell vaccines, inducing DC-CTL, TCR-T, and assessing the PD-1/CTLA4 prognosis.

### Availability and requirements

- Project name: PSSMHCpan
- Project home page: https://github.com/BGI2016/PSSMHCpan
- Operating system: Platform independent
- Programming language: Perl
- Other requirements: ActivePerl 5.8
- License: MIT

### Availability of supporting data and materials

The supporting data from this study are available in the PSSMHCpan homepage [44] and further supporting data, including snapshots of code, are available in the *GigaScience* database, GigaDB [45].

### Additional files

**Additional file 1.** Supplementary tables for supporting the analysis part.

**Additional file Table S1.** is the list of HLA class I alleles and corresponding peptide length for allele-specific and pan-specific prediction. Table S2 is 10-fold cross-validation results of alleles-specific prediction of PSSMHCpan, and the same validation on NetMHC, NetMHCpan, PickPocket, Nebula, sNebula, and SMM. Table S3 is the pan-specific prediction results. Table S4 is the prediction results of the independent dataset evaluation. Table S5 is the validation results of 9mer peptides bound to HLA-A*0201. The first column of “size of training database” represents the number of binder in training PSSMs.

### Competing interests

The authors declare no competing financial interests.

### Author contributions

GL, DL, ZL, BL, YH, JW, and HY conceived of study and designed the project. GL, DL, and ZL performed software development, computational analyses, and prepared figures. SQ and WL performed pan-cancer neoantigen analysis. GL, BL, and KM wrote the manuscript. CC, NY, HL, ZC, XS, LC, XZ, JW, and HY helped to revise the manuscript. All authors read and approved the final manuscript.

## Supplementary Material

GIGA-D-16-00055_Original_Submission.pdf

GIGA-D-16-00055_Revision_1.pdf

GIGA-D-16-00055_Revision_2.pdf

Response_to_reviewer_comments_Original_Submission.pdf

Response_to_reviewer_comments_Revision_1.pdf

Reveiwer_2_Report_(Revision_1).pdf

Reviewer_2_Report_(Original_Submission).pdf

Reviewer_3_Original_Submission_(Attachement).pdf

Reviewer_3_Report_(Original_Submission).pdf

Reviewer_3_Report_(Revision_1).pdf

Reviewer_3_Report_(Revision_2).pdf

Reviewer_3_Revision_1_(Attachement).pdf

Supplemental material **Additional file 1.** Supplementary tables for supporting the analysis part.
**Additional file Table S1.** is the list of HLA class I alleles and corresponding peptide length for allele-specific and pan-specific prediction. Table S2 is 10-fold cross-validation results of alleles-specific prediction of PSSMHCpan, and the same validation on NetMHC, NetMHCpan, PickPocket, Nebula, sNebula, and SMM. Table S3 is the pan-specific prediction results. Table S4 is the prediction results of the independent dataset evaluation. Table S5 is the validation results of 9mer peptides bound to HLA-A*0201. The first column of “size of training database” represents the number of binder in training PSSMs.
