# Supplementary material for: PSSMHCpan: a novel PSSM-based software for predicting class I peptide-HLA binding affinity
Source: Gigascience. 2017 Mar 15;6(5):1–11. doi: 10.1093/gigascience/gix017 (PMC5467046; doi:10.1093/gigascience/gix017)
Supplement: GIGA-D-16-00055_Original_Submission.pdf [file gix017_GIGA-D-16-00055_Original_Submission.pdf]

Manuscript

Click here to view linked References

Click here to download Manuscript PSSMHCpan a novel PSSM based software for predicting class I peptide-HLA

PSSMHCpan: a novel PSSM based software for predicting class I peptide-HLA binding affinity

Geng Liu†[1], Dongli Li†[1], Si Qiu[1, 2], Wenhui Li[1], Kun Ma[1], Jian Wang[1, 3], Huanming Yang[1, 3], Yong Hou*[1, 4], Bo Li*[1, 5]

[1] BGI-Shenzhen, Shenzhen 518083 China

[2] BGI Education Center, University of Chinese Academy of Sciences, Shenzhen 518083, China

[3]James D. Watson Institute of Genome Sciences, Hangzhou 310058, China

[4]Department of Biology, University of Copenhagen, Denmark

[5]BGI-Forensics, Shenzhen 518083, China

†These authors contributed equally to this work as first authors.

*To whom correspondence should be addressed. Tel: (86) 18680679919; Fax: (86) 0755-25273620; E-mail: libo@genomics.cn (B.L.). Correspondence may also be addressed. E-mail: houyong@genomics.cn (Y.H.).

Abstract

Background: Predicting peptides binding affinity with human leukocyte antigen (HLA) is a crucial step in developing powerful antitumor vaccine for cancer immunotherapy. Currently available methods work reasonably well in predicting peptide binding affinity with HLA-A*0201, HLA-A*0101, and HLA-B*0702 in terms of sensitivity and specificity. However, it is unknown whether these methods can also predict well with other HLA alleles that are present in majority of human populations.

Result: Here we present a Position Score Specific Matrix (PSSM) based software called PSSMHCpan to accurately and efficiently predict peptide binding affinity with a broad coverage of HLA class I alleles. By analyzing 10 cross-validations on training database of 87 HLA alleles and an independent

dataset with NetMHC-4.0, NetMHCpan-3.0, PickPocket, and PSSMHCpan, we found that PSSMHCpan is substantially better than the other three methods with accuracy ACC of 0.92 and sensitivity of 0.87, as compared to 0.85, 0.85, 0.72 in 10 cross-validations and 0.73, 0.79, 0.75 in the independent dataset evaluation. In addition, PSSMHCpan is more than 763 times faster than other three methods to predict neoantigens from a breast tumor sample. Finally we built a neoantigen prediction pipeline and identified 117,017 neoantigens from 467 cancer samples of diverse cancers from TCGA.

Conclusion: PSSMHCpan is superior to currently available methods in predicting peptide binding affinity with a broad coverage of HLA class I alleles.

Key words: Antitumor vaccine, peptide-HLA binding affinity, PSSMHCpan, neoantigen.

Background

Cancer immunotherapy has been proved to be a promising strategy that enhances the strengths of the immune system of cancer patients to fight cancer in recent years. This strategy exploits the fact that surface of cancer cells have a variety of tumor antigens (i.e. peptides of 8-13 residues in lengths) coming from various kinds of mutated proteins cleaved by the proteasomes intracellular. These peptides are bound to HLA class I allelic specific molecules, forming peptide-HLA complexes which are presented to T cell receptors (TCRs). If TCRs can recognize the complexes on the surface of cancer cells, cytotoxic T lymphocytes (CTLs) will destroy cancer cells. Cancer cells are highly heterogeneous in terms of morphological, phonotypical and genetic profiles. Cancer cells of different tumors and within the same tumor could present hundreds of different types of peptides. The immune system of cancer patients could only recognize small populations of cancer cells. In order to enhance the power of the CTLs to recognize and eradicate as many cancer cells as possible, one strategy is to vaccinate cancer patients with complex antitumor peptides. The first step to develop powerful antitumor vaccines is to predict peptide binding affinity with HLA class I allele.

In order to predict peptide binding affinity with HLA class I allele, four types of methods have been developed, including structure based methods, machine learning based methods, PSSM based methods [1] and combined methods. The structure based methods predict peptide binding affinity by calculating the minimum free energy of peptide-HLA complex [2], which allows us to understand the peptide-HLA binding affinity at the structure level. However, the predicting speed of this types of methods is extremely slow, and inaccurate due to limited number of available crystal structures [3]. The machine learning based methods predict peptide binding affinity by learning a function that maps a given peptide to binding affinity based on available known bound peptides (binders). These methods can accurately predict peptides with specific HLA alleles of HLA-A*0201, HLA-A*0101, and HLA-B*0702 [4, 5]. Hence, they are widely used in many studies [6-8]. Thus far, many methods of machine learning have been developed, including support vector machine based method MHC2PRED [9], hidden markov model based method S-HMM [10], artificial neural network based method NetMHC [11, 12], and pan-specific method NetMHCpan [13-15]. However, machine learning methods cannot accurately predict peptide binding affinity with a broad range of HLA class I allelic coverage. Further, they are inefficient in predicting peptides from a large amount of sequencing data. The PSSM based methods predict peptide binding affinity by building a matrix from multiple peptides alignment results that represent the motif information (i.e. the binding anchor). These methods have a faster predicting speed because linear computational complexity of PSSM is much lower than nonlinear computational complexity of structure and machine learning based methods. Based on the mechanism of PSSM, several software have been developed such as PickPocket [16], SVMHC [17] and nHLAPred [18]. However the accuracy of current software is less than machine learning based methods [16]. Recently, in order to predict peptide-HLA binding affinity more accurately, scientists from several

groups combined different methods to develop new software including NetMHCcons [19], IEDB [20] and HLaffy [21]. Although these combined methods indeed have shown a better predictive performance as compared to individual methods, their predictive accuracy are still not satisfactory, especially in clinical applications [22]. In order to develop more effective immunotherapy, it is necessary to develop better software that can more accurately and efficiently predict peptide binding affinity with a broad coverage of HLA class I alleles.

Here, we present a novel software called PSSMHCpan. We designed this software based on the PSSM mechanism and using a more comprehensive training database containing 63,099 peptide-HLA pairs to allele-specifically predict peptide binding affinity with HLA class I allele. In order to predict peptide binding affinity with a broad coverage of HLA class I alleles, we induce a simple but powerful pan-specific prediction approach based on the similarity of HLA protein sequences. We show that PSSMHCpan can predict peptide binding affinity with a broad HLA class I allelic coverage of at least 87 types more accurately and efficiently than other available methods in 10 cross-validations and independent dataset evaluation. Based on PSSMHCpan, we built a prediction pipeline to identify neoantigens in 467 TCGA tumor samples across 10 types of cancers.

Methods

PSSM is represented as a motif of multiple sequence alignment result [23]. The basic principle of PSSMHCpan is that peptides that bind to a specific HLA allele possess the motif information that can be studied by PSSM. We propose the PSSMHCpan in two novel aspects. Firstly, we construct a comprehensive training database to build allele-specific PSSMs for predicting peptide binding affinity with characterized HLA class I allele (with binders in training database). Secondly, we use the

similarity of HLA sequences to induce a simple but powerful pan-specific prediction approach based on our hypothesis below to predict peptide binding affinity with uncharacterized HLA class I allele (without binder in training database). It is well known that peptides on the cell surface are bound to the floor of the peptide-binding groove that is in the central region of the α1/α2 heterodimer (a molecule composed of two non-identical subunits) of HLA protein sequences [24]. By analyzing the sequences of HLA proteins, we noticed that HLA protein sequences are highly similar among different HLA alleles (Figure 1), and that peptides bound to similar HLA alleles have similar binding affinity according to predictive value of IC50. Thereby, we hypothesize that since different HLA protein sequences are similar, the peptide binding affinity with different HLA alleles should be similar too. Based on this hypothesis and the PSSM mechanism, we design the software PSSMHCpan as following three steps: PSSM construction, allele-specific prediction, and pan-specific prediction. The flowchart of PSSMHCpan is shown in Figure 2.

PSSM construction

We define PSSM as a matrix of M rows (Amino acid; M=20) and N columns (Length; N=8~25). Each element P_{ai} in the matrix is the likelihood of a given character (amino acid) at its position. We calculate the element P_{ai} through the following function,

$$P_{ai} = log\frac{F_{ai} + \omega}{BG_a}$$

Where $\mathrm{F_{ai}}$ denotes the frequency of amino acid a at position i ; $\mathrm{BG_a}$ denotes the background frequency of amino acid *a* from UniProt database [25]; and ω is a random value (ranging from 0 to 1) generated from Dirichlet distribution [26].

Allele-specific prediction

To qualitatively predict peptide binding affinity with characterized HLA allele, we define a *binder_score* as a sum of the corresponding values of each amino acid of a given peptide at each position in the corresponding allele-specific PSSM.

$$\text{binder\_score} = \frac{\sum_{i=1}^{N} P_{ai}}{N}$$

We consider a peptide with *binder_score > 0* as a binder. The higher *binder_score* that a peptide has, the higher binding affinity this peptide would have.

We convert a binding affinity score (*binder_score*) into an IC50 value as follows:

$$\text{IC50} = 50000^{Max-\text{binder\_}score / Max-Min}$$

Where Max and Min denote the maximum and the minimum *binder_score*, respectively. We consider a peptide with *IC50 < 500nM* as a binder and a peptide with *IC50 < 50nM* as a strong binder.

Pan-specific prediction

Firstly, we construct a library of HLA similar weight (Button panel in Figure 2) that contains pairs of characterized and uncharacterized HLA alleles, and each pair has a weight value. We determine a pair of characterized and uncharacterized HLA alleles by using the BLOSUM62 [27] based BLAST alignment results of HLA protein sequences, and assign the alignment score as the weight value. We also extracted the nearest distance of HLA alleles from NetMHCpan-3.0 [15] as a pair of characterized and uncharacterized HLA alleles and assigned a constant as the weight value.

Secondly, we qualitatively predict the binding affinity of a given peptide with uncharacterized HLA allele with an $IC50_{un}$ value which is calculated as below:

$$IC50_{un} = \frac{\sum_{i=1}^{S} w_i * \text{IC50}_i}{\sum_{i=1}^{S} w_i}$$

Where S denotes the sum of characterized HLA alleles that pair up the specific uncharacterized HLA allele according to the library of HLA similar weight. w_i and $IC50_i$ denote the weight value and the allele-specific prediction result of peptide binding affinity with HLA allele *i*. We also consider a peptide with $IC50_{un} < 500nM$ as a binder, and a peptide with $IC50_{un} < 50nM$ as a strong binder.

Data Description

We collected our training database of HLA class I binders from the following resources: the Immune Epitope Database and Analysis Resource (IEDB) [28], IEDB benchmark [29], SYFPEITHI [30], MHCBN [31], and in-house experimental epitopes. After removing duplications, we obtained 64,677 peptide-HLA pairs that cover 162 HLA alleles (Table 1). We only selected HLA alleles that consist of at least 10 binders with a fixed length. Finally, we built 241 PSSMs for allele-specific prediction of peptide binding affinity with 123 HLA class I alleles (Additional file 1: Table S1).

Table 1 Summary of training database.

Database	IEDB	IEDB benchmark	SYFPEITHI	MHCBN	Combined	Training database
**HLA alleles**	166	95	109	103	162	123
**Binders**	54,272	40,930	3,329	4,070	64,677	63,099

We collected 64 uncharacterized HLA class I alleles that cannot be predicted with NetMHC-4.0 but can be predicted with NetMHCpan-3.0. We extracted 2064 binders that bind to the 64 uncharacterized HLA alleles from our training database as a dataset for pan-specific evaluation.

To construct a library of HLA weight similarity, we collected 657,397 pairs of characterized and uncharacterized HLA class I alleles from 13,957 HLA protein sequences in IMGT/HLA (Release 3.23.0) [32], and 2800 pairs from the nearest distance of HLA alleles in NetMHCpan-3.0, respectively.

After removing duplications, we retained 657,930 pairs for pan-specific prediction of peptide binding affinity with 4,778 HLA class I alleles (Additional file 1: Table S1).

We also collected an independent dataset of binders from the Peptide Database of Cancer Immunity [33]. Then we selected 285 binders that cover 38 HLA alleles of HLA-A, HLA-B, HLA-C, including 35 from tumor antigens resulting from mutations, 91 from shared tumor-specific antigens, 63 from differentiation antigens and 96 from antigens overexpressed in tumors. After removing duplications, we retained 273 binders for validation.

To detect pan-cancer neoantigens, we obtained somatic mutations of 467 TCGA cancer samples across 10 cancer types (Table 2) from GDC data portal (https://gdc-portal.nci.nih.gov/) and the RSEM gene expression data of these tumors and their corresponding normal samples from FireBrowse (http://firebrowse.org/). We also obtained the tumor RNASeq aligned bam files from dbGAP.

Table 2 Summary of 467 cancer samples from TCGA cohort.

Cancer type	Patient #	Cancer type	Patient #
BLCA	19	LIHC	47
BRCA	93	LUAD	57
COAD	16	PRAD	43
HNSC	39	STAD	28
KIRC	67	THCA	58

Analyses

Evaluation of peptide binding affinity prediction with a broad HLA class I allelic coverage

In order to evaluate the allele-specific prediction accuracy of PSSMHCpan with a broad HLA class I allelic coverage, we performed 10 cross-validations on training data of 87 HLA class I alleles that contain at least 12 binders. We generated non-binders randomly with the same number of binders, and performed allele-specific prediction of peptide-HLA binding affinity using our PSSMHCpan, and the two well-known and currently considered as the best software for peptide-HLA binding affinity prediction NetMHC-4.0 and NetMHCpan-3.0 [4], and with the latest reported PSSM based software PickPocket, respectively. We found that the performance of the four software appeared similar in terms of the average area under receiver operating characteristic curve (AUC) with the HLA alleles of HLA-A*0101, HLA-A*0201, and HLA-B*0702 (Additional file 1: Table S2). However, in terms of the prediction accuracy ACC ($ACC = \frac{TP+TN}{TP+FP+TN+FN}$, where TP, FP, TN and FN, represent true-positive, false-positive, true-negative and false-negative) under the cutoff at 500nM, PSSMHCpan is larger than NetMHC-4.0, NetMHCpan-3.0 and PickPocket (Table 3), suggesting that the PSSMHCpan delivers more accurate than the other three software in predicting peptide binding affinity with the HLA alleles of HLA-A*0101, HLA-A*0201, and HLA-B*0702 at 500nM. We also noticed that although the overall AUC of PSSMHCpan is slightly larger than that of any of the software with the rest HLA class I alleles (ranging from 1% to 2%; Figure 3a), the ACC of PSSMHCpan is much larger than those of other three software (ranging from 7% to 20%). By comparing the ACC of each HLA allele with a fixed peptide length among the four software, we found that the median ACC of PSSMHCpan is significantly larger than other three software (*P* <0.01, *Paired T test*; Figure 3b).

Table 3 Assessments (ACC values) of four software to predict peptide binding affinity with three HLA alleles.

	**A*0101 9mer**	**A*0201 9mer**	**B*0702 9mer**	**A*0101 10mer**	**A*0201 10mer**	**B*0702 10mer**
**PSSMHCpan**	**0.96**	**0.88**	**0.91**	**0.96**	**0.92**	**0.96**
**NetMHC-4.0**	0.86	0.86	0.87	0.86	0.88	0.90
**NetMHCpan-3.0**	0.85	0.86	0.87	0.83	0.87	0.88
**PickPocket**	0.65	**0.88**	0.85	0.53	0.89	0.81

Considering a one-time 10 cross-validation of randomly selection and non-binders construction might produce biased results, we repeated another five times of 10 cross-validations, and found that (Table 4) the standard deviations (SD) of AUCs are ≤ 0.0005, indicating no bias in the 10 cross-validation.

Table 4 The AUC and SD values in 5 times 10 cross-validations.

Time	PSSMHCpan	NetMHC-4.0	NetMHCpan-3.0	PickPocket
**1**	0.9693	0.9623	0.965	0.9494
**2**	0.9703	0.9633	0.9661	0.9507
**3**	0.9703	0.9633	0.9661	0.9506
**4**	0.9699	0.9632	0.966	0.9505
**5**	0.9699	0.9633	0.9657	0.9506
**SD**	0.0004	0.0004	0.0005	0.0005

To evaluate our pan-specific prediction, we retrained PSSMs without binders from the dataset for pan-specific evaluation. And then we predicted binders from the dataset for pan-specific evaluation and 2,064 randomly constructed non-binders by PSSMHCpan. Although the AUC of PSSMHCpan (0.93) is slightly lower than those of NetMHCpan-3.0 and PickPocket (0.96; Figure 3c; Additional file 1: Table S3), the ACC of PSSMHCpan (0.86) is much larger than those two software (0.75 and 0.73). By comparing the allele-specific prediction and pan-specific prediction of 3,408 correctly predicted peptides from the dataset for pan-specific evaluation, we found a high correlation between allele-specific and pan-specific prediction (Pearson' rho=0.89, P<0.01; Figure 3d), suggesting that our PSSMHCpan can quantitatively predict peptide-HLA binding affinity with profound accuracy.

We compared the performance of our PSSMHCpan with the latest software HLaffy developed by Mukherjee et al (2016) using the same peptides from the MHCBN. We removed all the binders from MHCBN in our training database and retrained our PSSMs with the rest of binders. Because the number of non-binders is much smaller than that of the binders in MHCBN, we only used binders to evaluate and calculated the prediction accuracy by sensitivity ($\mathrm{Sen} = \frac{TP}{\mathrm{TP+FP}}$). We found that our PSSMHCpan correctly detected 1309 binders, while HLaffy correctly detected 1179 binders (Table 5).

Table 5 Assessments of PSSMHCpan and HLaffy. The prediction of HLaffy was performed on webserver (http://proline.biochem.iisc.ernet.in/HLaffy/).

Allele	PSSMHCpan	HLaffy
HLA-A*0201	**100.00%**	91.99%
HLA-A*0203	**100.00%**	93.22%

HLA-A*0206	**100.00%**	93.44%
HLA-A*0301	**100.00%**	83.93%
HLA-A*1101	**100.00%**	96.00%
HLA-A*2402	**100.00%**	76.60%
HLA-A*3301	**100.00%**	83.33%
HLA-A*6801	**100.00%**	94.12%
HLA-A*6802	**95.45%**	72.73%
HLA-B*0702	**100.00%**	87.88%
HLA-B*3501	**99.16%**	89.08%
HLA-B*5301	**100.00%**	91.84%
HLA-B*5401	**100.00%**	88.10%
All	**99.85%**	89.93%

Evaluation of peptide binding affinity prediction with an independent dataset

Considering cross validation might overestimate prediction accuracy, we reevaluated PSSMHCpan, NetMHC-4.0, NetMHCpan-3.0 and PickPocket with an independent dataset containing 273 non-duplicated experimental binders from the Peptide Database of Cancer Immunity. If a peptide binds to any 4-digital HLA allele that belong to the given 2-digital HLA allele with a predicting binding affinity IC50 less than 500nM, we considered as binder. Totally, 245 of 273 (90%) binders were identified with the four software. Of the 245 binders identified, PSSMHCpan, NetMHC-4.0, NetMHCpan-3.0 and PickPocket identified 237, 199, 216, and 204, respectively (Figure 4; Additional file 1: Table S4), again indicating that PSSMHCpan can predict more binders than either NetMHC-4.0, NetMHCpan-3.0, or PickPocket can.

Evaluation of the software efficiency

As whole genome sequencing (WGS) and whole exome sequencing (WES) of cancer genome data are rapidly increasing, there is an urgent need to develop software that can quickly identify neoantigens from cancer genome data. To compare the efficiency of PSSMHCpan, NetMHC-4.0, NetMHCpan-3.0

and PickPocket (Table 6), we first calculated the predicting speed of 10-cross validation on training database with 87 HLA class I alleles and found that PSSMHCpan is much faster than other three. We then used each software to independently predict binding affinity of the same set of 661,263 peptides generated from a breast tumor sample containing 3062 somatic mutations and 6 HLA class I alleles. We found that it took about 6 seconds for PSSMHCpan to complete the analysis. In contrast, NetMHC-4.0, took 3.61 hours, NetMHCpan-3.0 took 28.63 hours, and PickPocket took 1.34 hours to complete the analysis. In general, PSSMHCpan are not only more accuracy but also faster than other methods.

Table 6 The predicting speed (CPU time) of the four software. The fastest ones were marked in bold.

Methods	10-cross validations	Breast tumour neoantigens prediction
**PSSMHCpan**	**18.40s**	**6.34s**
**NetMHC-4.0**	1056.83s	13001.57s
**NetMHCpan-3.0**	5371.16s	103060.24s
**PickPocket**	282.83s	4839.63s

CPU time was measured by second (s).

Pan-cancer neoantigens

To identify neoantigens that can be used as candidate markers to develop antitumor vaccine, we develop a neoantigen prediction pipeline to determine what types of mutated peptides in cancer cells could be brought to the cell surface by HLAs based on somatic small mutations (SSMs). In order to maximize prediction accuracy, we include PSSMHCpan, NetMHC-4.0, NetMHCpan-3.0 and PickPocket into our pipeline to detect neoantigens in TCGA tumor samples as following (Figure 5a). We first annotate missense SSMs including single nucleotide variants (SNVs), insertions and deletions

(InDels) with ANNOVAR [34] to create a list of tumor-specific peptides (8-13) with an in-house script. After HLA alleles are predicted with Seq2HLA [35], we predict neoantigens with PSSMHCpan, NetMHC-4.0, NetMHCpan-3.0 and PickPocket, respectively. Finally, we select a list of neoantigens that meet the following conditions: 1) Predicting as binders (IC50<500nM) by at least 2 software and taking the median value of IC50 as final result; 2) The IC50 value of a given SNV-derived neoantigen must be smaller than that of its corresponding wile type (WT) peptide [36]. Using this pipeline, we analyzed the neoantigens across 10 cancer types from TCGA cohort.

Totally we identified 117,017 neoantigens from 467 TCGA cancer samples. We calculated the number of neoantigens per SSM in different types of cancer and observed that STAD, PRAD and BRCA had the highest neoantigens with 2.54, 1.52 and 1.43 per SNV, respectively (Figure 5b), whereas the highest neoantigens per InDel were 2.76, 2.59 and 2.34 in PRAD, STAD and KIRC, respectively (Figure 5c). We also compared the neoantigen loads (number of neoantigens per sample) across 10 cancer types and found that STAD, COAD and BLCA tumors had the highest neoantigen loads with median values of 302, 182 and 163, while the THCA tumors had a lowest median neoantigen load of 30 (Figure 5d).

On average we identified 251 neoantigens in each tumor. We then investigated whether the expression level of HLA class I would be increased in cancer cells to bind neoantigens. Indeed, by looking at the mRNA expression in 467 TCGA tumor samples and their paired normal tissues, we found that the expression of HLA class I was markedly elevated in most tumors (Figure 5e). Since the amount of neoantigens differs substantially among different tumors, we examined whether the number of neoantigens was correlated with HLA class I expression level in each tumor. However, we did not find a correlation between the number of neoantigens and the HLA class I expression levels in tumors

(Pearson' rho=-0.05, *P*=0.33).

Discussion

Designing antitumor vaccine requires predicting peptide-HLA binding affinity with high accuracy. In this article, we have presented a novel software PSSMHCpan that allows us to predict peptide binding affinity with a broad coverage of HLA class I alleles. By comparing our PSSMHCpan with the most popular machine learning based methods NetMHC-4.0, NetMHCpan-3.0 and the most recently published PSSM based method PickPocket, we demonstrated that overall our PSSMHCpan is substantially better than the other three in predicting peptide-HLA binding affinity, in terms of accuracy and efficiency.

In recent years, PSSM based methods to predict peptide-HLA binding affinity were gradually replaced by machine learning based methods that are believed to have reliable accuracy and larger data prediction capability [3]. However, by comparing our PSSMHCpan with machine learning based methods NetMHC-4.0 and NetMHCpan-3.0, we show that our PSSMHCpan exhibits a higher predicting accuracy than NetMHC-4.0 and NetMHCpan-3.0, respectively. In terms of data prediction capability, PSSMHCpan can allele-specifically and pan-specifically predict peptides that bind to 241 and 4778 HLA class I alleles, while NetMHC-4.0 and NetMHCpan-3.0 can only predict 89 and 2924 HLA class I alleles, respectively. Furthermore, the PSSMHCpan displays much higher prediction efficiency as compared to NetMHC-4.0 and NetMHCpan-3.0 (Table 6).

We noticed that the size of training database appeared to directly affect the prediction accuracy. A larger training database could improve the prediction accuracy of PSSMHCpan. For instance, the PSSMHCpan prediction accuracy ACC in predicting 9mer peptides bind to HLA-A*0101 and

HLA-B*5703 are 0.96 and 0.70. We found that in our training database, there are 813 binders for HLA-A*0101 and only 25 binders for HLA-B*5703, respectively. We believed that in order to improve the prediction accuracy, it is necessary to increase the size of training database.

Based on the evaluation results (Figure 4), we recognized that none of the available software is perfect and that in order to maximize the prediction accuracy, it is necessary to use multiple software. We then included PSSMHCpan, NetMHC-4.0, NetMHCpan-3.0 and PickPocket to build a neoantigen prediction pipeline that allowed us to detect 117,017 neoantigens in 467 TCGA tumor samples across 10 types of cancer. We believe that in order to provide actionable neoantigens that can be used in cancer immunotherapy, it requires more efforts to validate the function and immunogenicity of the predicted neoantigens experimentally.

In conclusion, our PSSMHCpan can predict peptide binding affinity with a broad coverage of HLA class I alleles more accurately and efficiently compared with currently most popular peptide binding affinity prediction software. Our PSSMHCpan can not only help develop personalized antitumor vaccines, but also has great potentials in other aspects of cancer immunotherapy including designing dendritic cell (DC) vaccines, inducing DC-CTL, TCR-T, and assessing the PD-1/CTLA4 prognosis.

Availability and requirements

- Project name: PSSMHCpan
- Project home page: https://github.com/BGI2016/PSSMHCpan
- Operating system: Platform independent
- Programming language: Perl
- Other requirements: ActivePerl 5.8

- License: OSI

Availability of supporting data and materials

The supporting data from this study will be hosted in the additional files and PSSMHCpan home page.

Additional file

Additional file 1: Supplementary tables for supporting the analysis part

Table S1 is the list of HLA class I alleles for allele-specific and pan-specific prediction. Table S2 is 10-cross validations results of alleles-specific prediction. Table S3 is the pan-specific prediction results. Table S4 is prediction results the independent dataset.

Competing interests

The authors declare no competing financial interests.

Authors' contributions

G. L., D. L, B. L. Y. H, J. W. and H. Y. conceived of study and designed the project. G. L. and D. L. performed software development, computational analyses and prepared figures. S. Q., W. L. performed pan-cancer neoantigen analysis. G. L., B. L. and K. M. wrote the manuscript.

Acknowledgements

We thank L. Goodman and M Dean for their constructive advices on the manuscript. We also thank Lei Ge and Yan Liang for their administrative supports. We would like to thank The Cancer Genome Atlas (TCGA), International Cancer Genome Consortium (ICGC), and Chinese Cancer Genome Consortium (CCGC) for letting us using the sequencing data. This project was supported financially with funds from the Shenzhen Science and Technology Program (JCYJ20150629114130814).

Reference

1. Liao WW, Arthur JW. Predicting peptide binding to Major Histocompatibility Complex molecules. Autoimmunity reviews. 2011;10(8):469-73. doi:10.1016/j.autrev.2011.02.003.
2. Schueler-Furman O, Altuvia Y, Sette A, Margalit H. Structure-based prediction of binding peptides to MHC class I molecules: application to a broad range of MHC alleles. Protein science : a publication of the Protein Society. 2000;9(9):1838-46. doi:10.1110/ps.9.9.1838.
3. Luo H, Ye H, Ng HW, Shi L, Tong W, Mendrick DL et al. Machine Learning Methods for Predicting HLA-Peptide Binding Activity. Bioinformatics and biology insights. 2015;9(Suppl 3):21-9. doi:10.4137/BBI.S29466.
4. Zhang GL, Ansari HR, Bradley P, Cawley GC, Hertz T, Hu X et al. Machine learning competition in immunology - Prediction of HLA class I binding peptides. Journal of immunological methods. 2011;374(1-2):1-4. doi:10.1016/j.jim.2011.09.010.
5. Nielsen M, Lundegaard C, Worning P, Lauemoller SL, Lamberth K, Buus S et al. Reliable prediction of T-cell epitopes using neural networks with novel sequence representations. Protein science : a publication of the Protein Society. 2003;12(5):1007-17. doi:10.1110/ps.0239403.
6. Carreno BM, Magrini V, Becker-Hapak M, Kaabinejadian S, Hundal J, Petti AA et al. Cancer immunotherapy. A dendritic cell vaccine increases the breadth and diversity of melanoma neoantigen-specific T cells. Science. 2015;348(6236):803-8. doi:10.1126/science.aaa3828.
7. Yadav M, Jhunjhunwala S, Phung QT, Lupardus P, Tanguay J, Bumbaca S et al. Predicting immunogenic tumour mutations by combining mass spectrometry and exome sequencing. Nature. 2014;515(7528):572-6. doi:10.1038/nature14001.
8. Walter S, Weinschenk T, Stenzl A, Zdrojowy R, Pluzanska A, Szczylik C et al. Multipeptide immune response to cancer vaccine IMA901 after single-dose cyclophosphamide associates with longer patient survival. Nature medicine. 2012;18(8):1254-61. doi:10.1038/nm.2883.
9. Lata S, Bhasin M, Raghava GP. Application of machine learning techniques in predicting MHC binders. Methods in molecular biology. 2007;409:201-15. doi:10.1007/978-1-60327-118-9_14.
10. Noguchi H, Kato R, Hanai T, Matsubara Y, Honda H, Brusic V et al. Hidden Markov model-based prediction of antigenic peptides that interact with MHC class II molecules. Journal of bioscience and bioengineering. 2002;94(3):264-70.
11. Lundegaard C, Lund O, Nielsen M. Prediction of epitopes using neural network based methods. Journal of immunological methods. 2011;374(1-2):26-34. doi:10.1016/j.jim.2010.10.011.
12. Andreatta M, Nielsen M. Gapped sequence alignment using artificial neural networks: application to the MHC class I system. Bioinformatics. 2016;32(4):511-7. doi:10.1093/bioinformatics/btv639.
13. Hoof I, Peters B, Sidney J, Pedersen LE, Sette A, Lund O et al. NetMHCpan, a method for MHC class I binding prediction beyond humans. Immunogenetics. 2009;61(1):1-13. doi:10.1007/s00251-008-0341-z.
14. Nielsen M, Lundegaard C, Blicher T, Lamberth K, Harndahl M, Justesen S et al. NetMHCpan, a method for quantitative predictions of peptide binding to any HLA-A and -B locus protein of known sequence. PloS one. 2007;2(8):e796. doi:10.1371/journal.pone.0000796.
15. Nielsen M, Andreatta M. NetMHCpan-3.0; improved prediction of binding to MHC class I molecules integrating information from multiple receptor and peptide length datasets. Genome medicine. 2016;8(1):33. doi:10.1186/s13073-016-0288-x.
16. Zhang H, Lund O, Nielsen M. The PickPocket method for predicting binding specificities for receptors based on receptor pocket similarities: application to MHC-peptide binding. Bioinformatics.

2009;25(10):1293-9. doi:10.1093/bioinformatics/btp137.

17. Donnes P, Kohlbacher O. SVMHC: a server for prediction of MHC-binding peptides. Nucleic acids research. 2006;34(Web Server issue):W194-7. doi:10.1093/nar/gkl284.

18. Bhasin M, Raghava GP. A hybrid approach for predicting promiscuous MHC class I restricted T cell epitopes. Journal of biosciences. 2007;32(1):31-42.

19. Karosiene E, Lundegaard C, Lund O, Nielsen M. NetMHCcons: a consensus method for the major histocompatibility complex class I predictions. Immunogenetics. 2012;64(3):177-86. doi:10.1007/s00251-011-0579-8.

20. Trolle T, Metushi IG, Greenbaum JA, Kim Y, Sidney J, Lund O et al. Automated benchmarking of peptide-MHC class I binding predictions. Bioinformatics. 2015;31(13):2174-81. doi:10.1093/bioinformatics/btv123.

21. Mukherjee S, Bhattacharyya C, Chandra N. HLaffy: estimating peptide affinities for Class-1 HLA molecules by learning position-specific pair potentials. Bioinformatics. 2016. doi:10.1093/bioinformatics/btw156.

22. Backert L, Kohlbacher O. Immunoinformatics and epitope prediction in the age of genomic medicine. Genome medicine. 2015;7(1):119. doi:10.1186/s13073-015-0245-0.

23. Xia X. Position weight matrix, gibbs sampler, and the associated significance tests in motif characterization and prediction. Scientifica. 2012;2012:917540. doi:10.6064/2012/917540.

24. Toh H, Savoie CJ, Kamikawaji N, Muta S, Sasazuki T, Kuhara S. Changes at the floor of the peptide-binding groove induce a strong preference for proline at position 3 of the bound peptide: molecular dynamics simulations of HLA-A*0217. Biopolymers. 2000;54(5):318-27. doi:10.1002/1097-0282(20001015)54:5<318::AID-BIP30>3.0.CO;2-T.

25. Apweiler R, Bairoch A, Wu CH, Barker WC, Boeckmann B, Ferro S et al. UniProt: the Universal Protein knowledgebase. Nucleic acids research. 2004;32(Database issue):D115-9. doi:10.1093/nar/gkh131.

26. Altschul SF, Gertz EM, Agarwala R, Schaffer AA, Yu YK. PSI-BLAST pseudocounts and the minimum description length principle. Nucleic acids research. 2009;37(3):815-24. doi:10.1093/nar/gkn981.

27. Styczynski MP, Jensen KL, Rigoutsos I, Stephanopoulos G. BLOSUM62 miscalculations improve search performance. Nature biotechnology. 2008;26(3):274-5. doi:10.1038/nbt0308-274.

28. Vita R, Overton JA, Greenbaum JA, Ponomarenko J, Clark JD, Cantrell JR et al. The immune epitope database (IEDB) 3.0. Nucleic acids research. 2015;43(Database issue):D405-12. doi:10.1093/nar/gku938.

29. Kim Y, Sidney J, Buus S, Sette A, Nielsen M, Peters B. Dataset size and composition impact the reliability of performance benchmarks for peptide-MHC binding predictions. BMC bioinformatics. 2014;15:241. doi:10.1186/1471-2105-15-241.

30. Schuler MM, Nastke MD, Stevanovikc S. SYFPEITHI: database for searching and T-cell epitope prediction. Methods in molecular biology. 2007;409:75-93.

31. Bhasin M, Singh H, Raghava GP. MHCBN: a comprehensive database of MHC binding and non-binding peptides. Bioinformatics. 2003;19(5):665-6.

32. Robinson J, Soormally AR, Hayhurst JD, Marsh SG. The IPD-IMGT/HLA Database - New developments in reporting HLA variation. Human immunology. 2016. doi:10.1016/j.humimm.2016.01.020.

33. Vigneron N, Stroobant V, Van den Eynde BJ, van der Bruggen P. Database of T cell-defined human tumor antigens: the 2013 update. Cancer immunity. 2013;13:15.

34. Wang K, Li M, Hakonarson H. ANNOVAR: functional annotation of genetic variants from high-throughput sequencing data. Nucleic acids research. 2010;38(16):e164. doi:10.1093/nar/gkq603.
35. Boegel S, Lower M, Schafer M, Bukur T, de Graaf J, Boisguerin V et al. HLA typing from RNA-Seq sequence reads. Genome medicine. 2012;4(12):102. doi:10.1186/gm403.
36. Hundal J, Carreno BM, Petti AA, Linette GP, Griffith OL, Mardis ER et al. pVAC-Seq: A genome-guided in silico approach to identifying tumor neoantigens. Genome medicine. 2016;8(1):11. doi:10.1186/s13073-016-0264-5.

FIGURE LEGENDS

Figure 1 Heat map of HLA protein sequence similarity. The larger the Z-Score, the more similar of the pair HLA protein sequences. It showed high similarity between different types of HLA alleles within the same gene locus.

Figure 2 Method of PSSMHCpan. The three mainly steps are shown in grey background.

Figure 3 Evaluation on broad HLA allelic coverage. (a) The allele-specific prediction evaluation results showed by ROC curse of PSSMHCpan, NetMHC-4.0, NetMHCpan-3.0 and PickPocket. This result was except 9mer and 10mer of HLA-A*0101, HLA-A*0201 and HLA-B*0702. The ACC, sensitivity and specificity at cutoff of 500nM were also shown. (b) The boxplot of individual ACC of particular HLA allele with fixed peptide length. Comparison between PSSMHCpan and other three methods were performed by using paired T test. "*" denotes $P<0.05$ and "**" denotes $P<0.01$. (c) The evaluation results showed by ROC curse of PSSMHCpan in pan-specific prediction, NetMHCpan-3.0 and PickPocket. The ACC, sensitivity and specificity at cutoff of 500nM were also shown. (d) Correlation analysis of peptide-HLA binding affinity result of IC50 value in log2 between allele-specific prediction and pan-specific prediction.

Figure 4 The evaluation result of the independent dataset. We denoted IC50<500nM was positive prediction.

Figure 5 Pan-cancer neoantigens. (a) The flow-char of neoantigen prediction pipeline. Software with

parameters using in the pipeline are shown in dashed procedure. (b) The distribution of neoantigens generated from each SNV across diverse cancers. (c) The distribution of neoantigens generated from each InDel across diverse cancers. (d) The distribution of neoantigen loads across 10 cancer types. The cancer types are sorted by median value of neoantigen loads. (e) The expression of HLA class I in tumor and corresponding normal samples.

Figure

Click here to download Figure Figure1.pdf

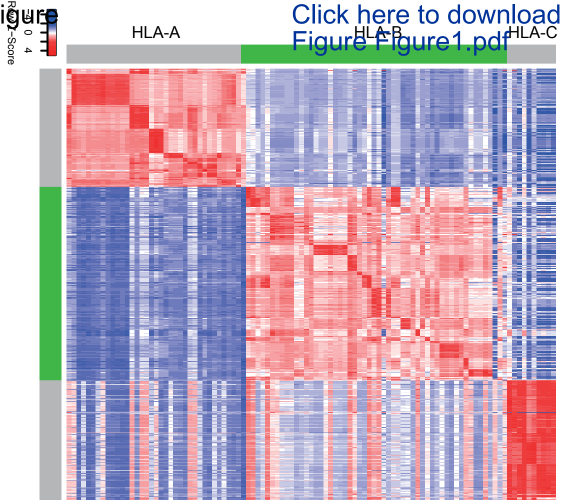

Figure

Click here to download Figure Figure2.pdf

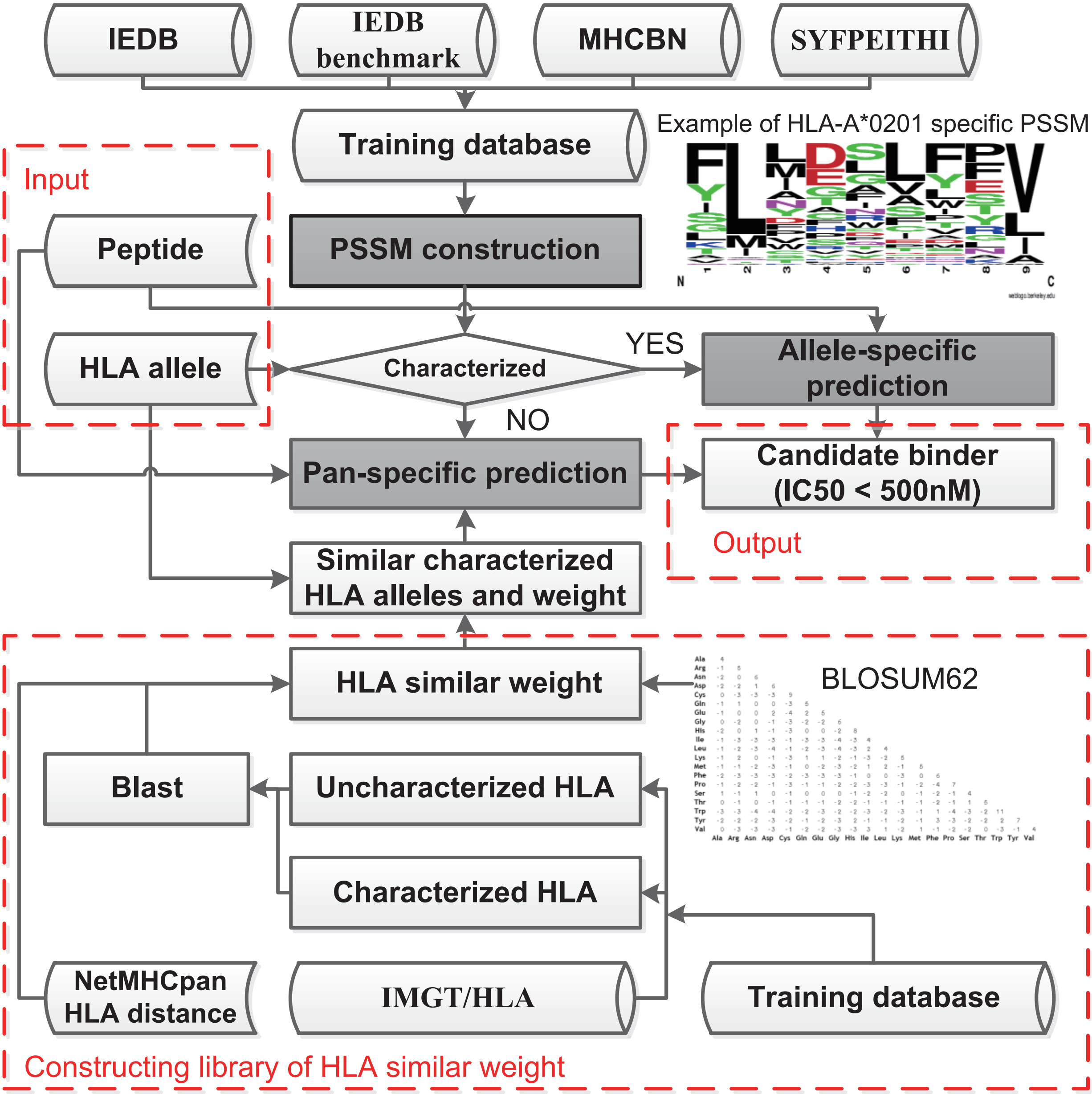

Figure

Click here to download Figure Figure3.pdf

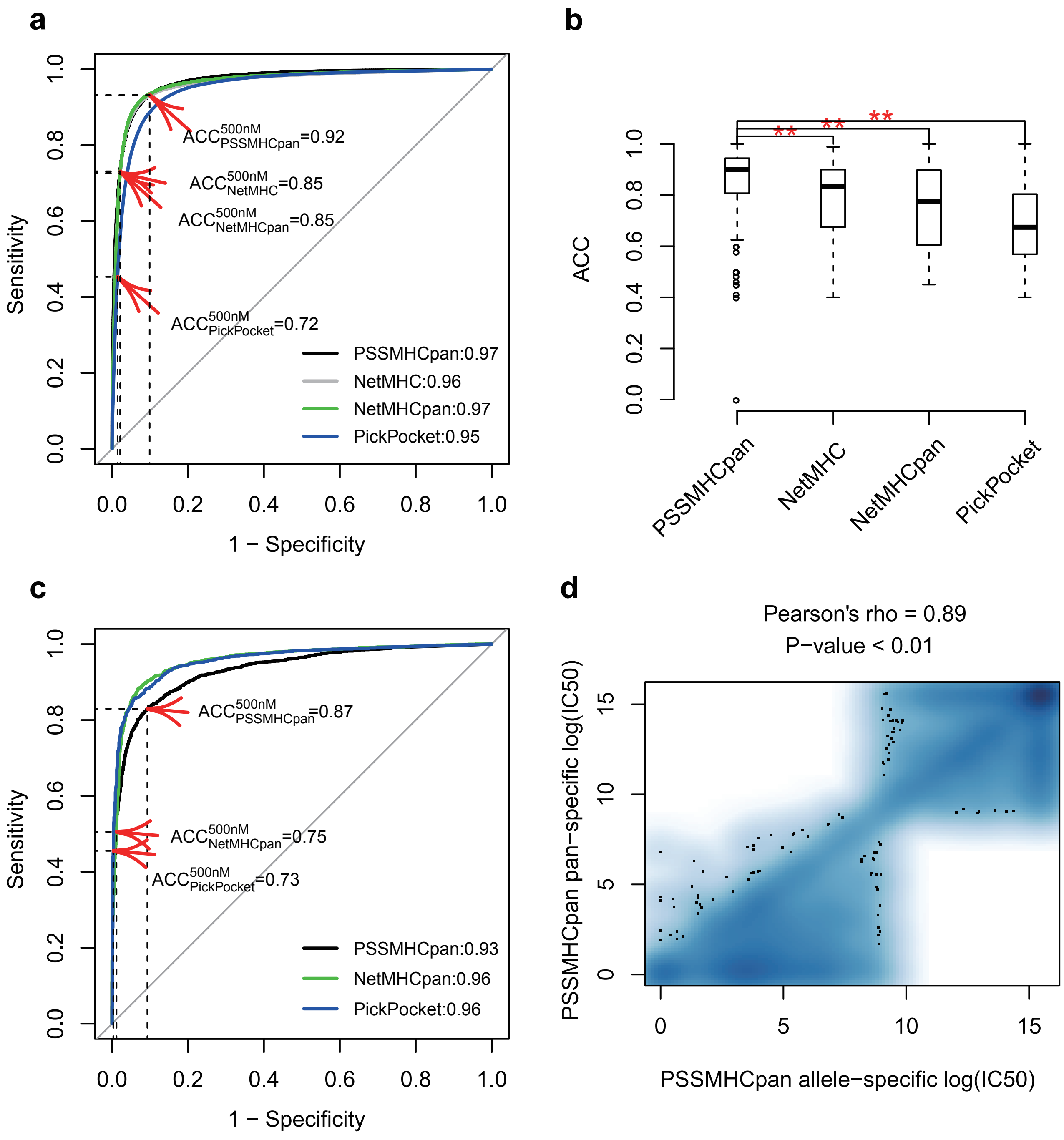

Figure
Click here to download Figure Figure4.pdf

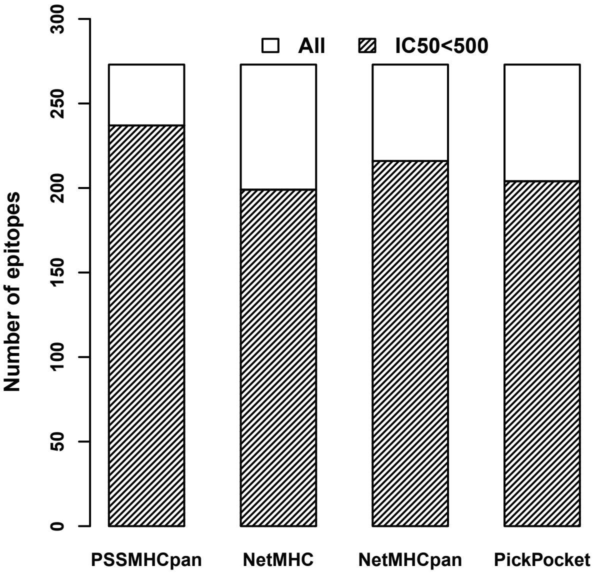

Figure

Click here to download Figure Figure5.pdf

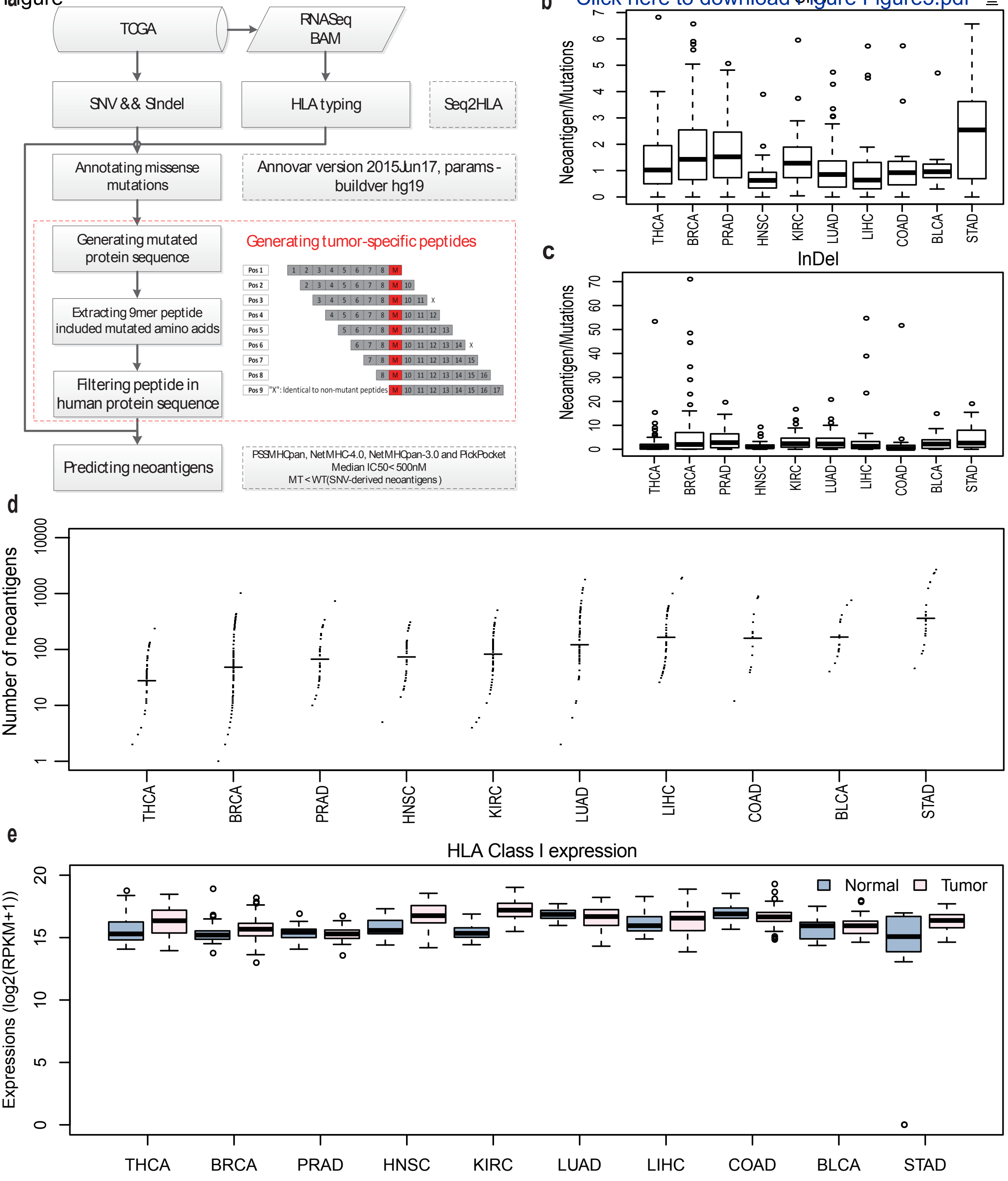

Click here to access/download
Supplementary Material
Additional file 1.xlsx
