# Supplementary material for: PSSMHCpan: a novel PSSM-based software for predicting class I peptide-HLA binding affinity
Source: Gigascience. 2017 Mar 15;6(5):1–11. doi: 10.1093/gigascience/gix017 (PMC5467046; doi:10.1093/gigascience/gix017)
Supplement: GIGA-D-16-00055_Revision_1.pdf [file gix017_GIGA-D-16-00055_Revision_1.pdf]

Manuscript

Click here to download Manuscript PSSMHCpan manuscript.docx

PSSMHCpan: a novel PSSM based software for predicting class I peptide-HLA binding affinity

Geng Liu[†1], Dongli Li[†1], Si Qiu[1, 2], Wenhui Li[1], Cheng-chi Chao[1, 3], Naibo Yang[1, 3], Handong Li[1, 3], Zhen Cheng[4], Xin Song[5], Le Cheng[1,6], Jian Wang[1, 7], Huanming Yang[1, 7], Yong Hou[*1, 8], Kun Ma[*1], Bo Li[*1, 9]

[1] BGI-Shenzhen, Shenzhen 518083 China

[2] BGI Education Center, University of Chinese Academy of Sciences, Shenzhen 518083, China

[3]Complete Genomics, Inc., 2071 Stierlin Court, Mountain View, CA 94043 USA

[4]Molecular Imaging Program at Stanford, Department of Radiology and Bio-X Program, Stanford University

[5]The third affiliated hospital of Kunming medical university (Tumor hospital of Yunnan province)

[6]BGI-Yunnan, Kunming 650000, China

[7]James D. Watson Institute of Genome Sciences, Hangzhou 310058, China

[8]Department of Biology, University of Copenhagen, Denmark

[9]BGI-Forensics, Shenzhen 518083, China

†These authors contributed equally to this work as first authors.

*To whom correspondence should be addressed. Tel: (86) 18680679919; Fax: (86) 0755-25273620; E-mail: libo@genomics.cn (Bo Li). Correspondence may also be addressed. E-mail: makun1@genomics.cn (Kun Ma) and houyong@genomics.cn (Yong Hong).

Abstract

Background: Predicting peptides binding affinity with human leukocyte antigen (HLA) is a crucial step in developing powerful antitumor vaccine for cancer immunotherapy. Currently available methods work quite well in predicting peptide binding affinity with HLA alleles such as HLA-A*0201, HLA-A*0101, and HLA-B*0702 in terms of sensitivity and specificity. However, quite a few types of HLA alleles that are present in majority of human populations including HLA-A*0202, HLA-A*0203, HLA-A*6802, HLA-B*5101, HLA-B*5301, HLA-B*5401 and HLA-B*5701 still cannot be predicted with satisfactory accuracy using currently available methods. Further, currently most popularly used methods for predicting peptides binding affinity are inefficient in identifying neoantigens from large quantity of whole genome and transcriptome sequencing data

Result: Here we present a Position Specific Scoring Matrix (PSSM) based software called PSSMHCpan to accurately and efficiently predict peptide binding affinity with a broad coverage of HLA class I alleles. We evaluated the performance of PSSMHCpan by analyzing 10-fold cross-validation on a training database containing 87 HLA alleles and obtained an average area under receiver operating characteristic curve (AUC) of 0.94 and accuracy ACC of 0.85. In an independent dataset (Peptide Database of Cancer Immunity) evaluation, PSSMHCpan is substantially better than popularly used NetMHC-4.0, NetMHCpan-3.0, PickPocket, Nebula, and SMM with a sensitivity of 0.90, as compared to 0.74, 0.81, 0.77, 0.24 and 0.79. In addition, PSSMHCpan is more than 197 times faster than NetMHC-4.0, NetMHCpan-3.0, PickPocket, sNebula and SMM when predicting neoantigens from 661,263 peptides from a breast tumor sample. Finally, we built a neoantigen prediction pipeline and identified 117,017 neoantigens from 467 cancer samples of various cancers from TCGA.

Conclusion: PSSMHCpan is superior to currently available methods in predicting peptide binding affinity with a broad coverage of HLA class I alleles.

Key words: Antitumor vaccine, peptide-HLA binding affinity, PSSMHCpan, neoantigen.

Background

Cancer immunotherapy has been proved to be a promising strategy that enhances the strengths of the immune system of cancer patients to fight cancer in recent years. This strategy exploits the fact that

surface of cancer cells have a variety of tumor antigens (i.e. peptides of 8-13 residues in lengths) coming from various kinds of mutated proteins cleaved by the proteasomes intracellular. These peptides are bound to HLA class I allelic specific molecules, forming peptide-HLA complexes which are presented to T cell receptors (TCRs). If TCRs can recognize these peptide-HLA complexes on the surface of cancer cells, cytotoxic T lymphocytes (CTLs) will destroy cancer cells. Cancer cells are highly heterogeneous in terms of morphological, phonotypical and genetic profiles. Cancer cells of different tumors and within the same tumor could present hundreds of different types of peptides. The immune system of cancer patients could only recognize small populations of cancer cells. In order to enhance the power of the CTLs to recognize and eradicate as many cancer cells as possible, one strategy is to vaccinate cancer patients with complex antitumor peptides. The first step to develop powerful antitumor vaccines is to predict peptide binding affinity with HLA class I allele.

In order to predict peptide binding affinity with HLA class I allele, four types of methods have been developed, including structure based methods, machine learning based methods, PSSM based methods [16] and combined methods. The structure based methods predict peptide binding affinity by calculating the minimum free energy of peptide-HLA complex [30], which allows us to understand the peptide-HLA binding affinity at the structure level. However, the predicting speed of this type of methods is extremely slow, and inaccurate due to limited number of available crystal structures [20]. The machine learning based methods predict peptide binding affinity by learning a function that maps a given peptide to areas with binding affinity based on available known bound peptides (binders). Because machine learning based methods can accurately predict peptides with specific HLA alleles such as HLA-A*0201, HLA-A*0101, and HLA-B*0702 [25, 41], they are frequently used in many studies [8, 37, 40]. Thus far, many machine learning based methods have been developed, including

support vector machine (SVM) based method MHC2PRED [15], hidden markov model (HMM) based method S-HMM [26], artificial neural network (ANN) based method NetMHC [2, 17], and pan-specific method NetMHCpan [11, 23, 24]. Although currently available tools can predict a number of HLA class I allelic coverage with appreciable AUCs, they cannot predict quite a few types of HLA alleles that are present in majority of human populations with satisfactory accuracy. For example, NetMHC, ARB, Nebula, sNebula and SMM only achieved the average predicted AUC of no more than 0.85 when they were used in predicting HLA-A*0202, HLA-A*0203, HLA-A*6802, HLA-B*5101, HLA-B*5301, HLA-B*5401 and HLA-B*5701 [19, 21, 27]. Further, these methods are inefficient in predicting large quantity of peptides generated from whole genome and transcriptome sequencing data because of their nonlinear computation complexity. In contrast, PSSM based methods predict peptide binding affinity by building a matrix from multiple peptides alignment that represent the motif information (i.e. the binding anchor). These methods can predict binding affinity fast because PSSM's linear computational complexity is much less complex than nonlinear computational complexity of structure-based and machine learning based methods. Based on the mechanism of PSSM, several software have been developed such as PickPocket [42], SVMHC [9] and nHLAPred [5]. However the predicting accuracy of these software is not as good as that of machine learning based methods [42]. Recently, in order to predict peptide-HLA binding affinity more accurately, scientists from several groups combined different methods to develop new software including NetMHCcons [13] and IEDB (combination of machine learning and PSSM) [34], and HLaffy (combination of structure and PSSM) [22]. Although these combined methods indeed have shown a better predictive performance as compared to individual methods, their predictive accuracy are still not satisfactory, especially in clinical applications [4]. In order to develop more effective immunotherapy, it is necessary to develop

better software that can more accurately and efficiently predict peptide binding affinity with a broad coverage of HLA class I alleles.

Here, we present a novel software called PSSMHCpan that can predict peptide binding affinity accurately and efficiently. We designed this software based on the PSSM mechanism and trained it with a larger database containing 63,099 peptide-HLA pairs which allow us to allele-specifically predict peptide binding affinity with HLA class I allele. In order to predict peptide binding affinity with a broad coverage of HLA class I alleles, we induce a simple but powerful pan-specific prediction approach based on the similarity of HLA protein sequences. We show that PSSMHCpan can accurately and efficiently predict peptide binding affinity with a broad HLA class I allelic coverage of at least 87 types in 10-fold cross-validation, and it performed better than other 5 software when evaluated with Peptide Database of Cancer Immunity dataset. Finally, we built a prediction pipeline to identify neoantigens in 467 TCGA tumor samples across 10 types of cancers.

Methods

PSSM is represented as a motif of multiple sequence alignment result [39]. The basic principle of PSSMHCpan is that peptides that bind to a specific HLA allele possess the motif information that can be studied by PSSM. We propose the PSSMHCpan in two novel aspects. Firstly, we construct a comprehensive training database and build allele-specific PSSMs for predicting peptide binding affinity with characterized HLA class I allele (with binders in training database). Secondly, we use the similarity of HLA sequences to induce a simple but powerful pan-specific prediction approach based on our hypothesis below, and predict peptide binding affinity with uncharacterized HLA class I allele (without binders in training database).

It is well known that peptides on the cell surface are bound to the floor of the peptide-binding groove that is in the central region of the α1/α2 heterodimer (a molecule composed of two non-identical subunits) of HLA protein sequences [33]. By analyzing the sequences of HLA proteins, we noticed that HLA protein sequences are highly similar among different HLA alleles (Figure 1), suggesting that peptides bound to similar HLA alleles have similar binding affinity according to predictive value of IC50. Thereby, we hypothesize that since different HLA protein sequences are similar, the peptide binding affinity with different HLA alleles should be similar too. Based on this hypothesis and the PSSM mechanism, we design the software PSSMHCpan as following three steps: PSSM construction, allele-specific prediction, and pan-specific prediction. The flowchart of PSSMHCpan is shown in Figure 2.

PSSM construction

We define PSSM as a matrix of M rows (Amino acid; M=20) and N columns (Length; N=8~25). Each element P_{ai} in the matrix is the likelihood of a given character (amino acid) at its position. We calculate the element P_{ai} through the following function,

$$P_{ai} = log\frac{F_{ai} + \omega}{BG_a}$$

Where F_{ai} denotes the frequency of amino acid *a* at position *i* from the training database; BG_a denotes the background frequency of amino acid *a* from UniProt database [3]; and ω is a random value (ranging from 0 to 1) generated from Dirichlet distribution [1].

Allele-specific prediction

To qualitatively predict peptide binding affinity with characterized HLA allele, we define a

binder_score as the sum of the corresponding values of each amino acid of a given peptide at each position in the corresponding allele-specific PSSM.

$$\text{binding\_score} = \frac{\sum_{i=1}^{N} P_{ai}}{N}$$

We consider a peptide with *binding_score > 0* as a binder according to the signal prediction of GeneID [10]. The higher *binding_score* that a peptide has, the higher binding affinity this peptide would have.

We convert a *binding_score* into an IC50 value as follows:

$$\text{IC50} = 50000^{\left(Max - \text{binding\_}score\right)/\left(Max - Min\right)}$$

Where Max and Min denote the maximum and the minimum values of *binding_score*, respectively. In this study, we assigned Max as 0.8 and Min as -0.8 based on our experience. According to the recommendation of IEDB [43], we consider a peptide with *IC50 < 500nM* as a binder and a peptide with *IC50 < 50nM* as a strong binder.

Pan-specific prediction

Firstly, we construct a library of HLA similar weight (Button panel in Figure 2) that contains pairs of characterized and uncharacterized HLA alleles, and each pair has a weight value. We determine a pair of characterized and uncharacterized HLA alleles by using BLOSUM62 [32] based BLAST alignment of HLA protein sequences, and assign the alignment score as the weight value. We also extracted the nearest distance of HLA alleles from NetMHCpan-3.0 [23] as a pair of characterized and uncharacterized HLA alleles and assigned a constant as the weight value.

Secondly, we qualitatively predict the binding affinity of a given peptide with uncharacterized HLA allele with an $IC50_{un}$ value which is calculated as below:

$$IC50_{un} = \frac{\sum_{i=1}^{S} w_i * IC50_i}{\sum_{i=1}^{S} w_i}$$

Where S denotes the sum of characterized HLA alleles that pair up the specific uncharacterized HLA allele according to the library of HLA similar weight. w_i and $IC50_i$ denote the weight value and the allele-specific prediction result of peptide binding affinity with HLA allele *i*. We also consider a peptide with $IC50_{un} < 500nM$ as a binder, and a peptide with $IC50_{un} < 50nM$ as a strong binder.

10-fold cross-validation

We apply 10-fold cross-validation [4] to evaluate the performance of peptide-HLA binding prediction as follows. Firstly, we randomly partitioned our collected data (See Data Description) into 10 subsets of nearly equal size, of which each consists of equal number of binders and non-binders. All the binders are experimentally verified, while the non-binders include experimentally verified ones from IEDB benchmark [14] and computer randomly constructed ones predicted as non-binders by any of the following four methods (PSSMHCpan, NetMHC-4.0, NetMHCpan-3.0 and PickPocket). We use computer constructed non-binders because currently available experimentally verified non-binders that meet our requirement only cover 50 class I HLA alleles. Subsequently, we performed 10 iterations of training and validation. In each iteration we use a different subset of data for validation, while the remaining 9 subsets for training.

Data Description

We collected our training database of HLA class I binders from the following resources: the Immune Epitope Database and Analysis Resource (IEDB) [36], IEDB benchmark [14], SYFPEITHI [31], MHCBN [6], and in-house experimental epitopes. After filtering out duplications and peptides with

abnormal amino acids which do not or rarely exist naturally, such as B, J, O, U, X and Z, we obtained 64,677 peptide-HLA pairs that cover 162 HLA alleles (Table 1). We only selected HLA alleles that consist of at least 10 binders with a fixed length. Finally, we built 241 PSSMs for allele-specific prediction of peptides with variable lengths (8~25 peptides) bound to 123 HLA class I alleles (Additional file 1: Table S1).

Table 1 Summary of training database.

Database	IEDB	IEDB benchmark	SYFPEITHI	MHCBN	Combined	Training database
**HLA alleles**	166	95	109	103	162	123
**Binders**	54,272	40,930	3,329	4,070	64,677	63,099

We collected 64 uncharacterized HLA class I alleles that cannot be predicted with NetMHC-4.0 but can be predicted with NetMHCpan-3.0. We extracted 2064 binders that bind to the 64 uncharacterized HLA alleles from our training database and same number of experimentally verified non-binders as a Dataset for Pan-specific evaluation (DP).

To construct a library of HLA weight similarity, we collected 690,497 pairs of characterized and uncharacterized HLA class I alleles from 13,957 HLA protein sequences in IMGT/HLA (Release 3.23.0) [29], and 2800 pairs from the nearest distance of HLA alleles in NetMHCpan-3.0, respectively. After removing duplications, we retained 691,031 pairs for pan-specific prediction of peptide binding affinity with 4,896 HLA class I alleles (Additional file 1: Table S1).

We also collected an independent dataset of binders from the Peptide Database of Cancer Immunity [35]. From this database, we selected 285 binders that cover 38 HLA alleles of HLA-A, HLA-B, HLA-C. After removing duplications, we retained 273 binders for validation.

To detect pan-cancer neoantigens, we obtained somatic mutations from 467 TCGA tumor samples

across 10 cancer types (Table 2) from GDC data portal (https://gdc-portal.nci.nih.gov/), and the RSEM gene expression data in these tumors and in their paired normal tissues from FireBrowse (http://firebrowse.org/). In addition, we also obtained the RNASeq aligned bam files from these tumors from dbGAP.

Table 2 Summary of 467 cancer samples from TCGA cohort.

Cancer type	Patient #	Cancer type	Patient #
BLCA	19	LIHC	47
BRCA	93	LUAD	57
COAD	16	PRAD	43
HNSC	39	STAD	28
KIRC	67	THCA	58

Analyses

Evaluation of peptide binding affinity prediction with a broad HLA class I allelic coverage

In order to evaluate the allele-specific prediction accuracy of PSSMHCpan with a broad HLA class I allelic coverage, we performed 10-fold cross-validation on training data of 87 HLA class I alleles that contain at least 12 binders, and obtained an average AUC of 0.94 and prediction accuracy ACC ($\mathrm{ACC} = \frac{\mathrm{TP+TN}}{\mathrm{TP+FP+TN+FN}}$, where TP, FP, TN and FN, represent true-positive, false-positive, true-negative and false-negative) of 0.85 under a cutoff of 500nM. We then used the same validation data to evaluate 6 popularly used software, i.e. NetMHC-4.0, NetMHCpan-3.0, PickPocket, Nebula [18], sNebula [19], and SMM [28], respectively. It is worth noting that the training data of these 6 software are from IEDB, IEDB benchmark, MHCBN, SYFPEITHI and so on [2, 18, 19, 23, 28, 42], which are largely

overlapped (over 65%) with the validation data in our 10-fold cross-validation analysis. Despite this substantial overlap (which will biasedly increase the AUC values for these software), we found that the AUC values of our PSSMHCpan are nearly equal or slightly lower than those of NetMHC-4.0, NetMHCpan-3.0, PickPocker and SMM, but nearly equal or higher than those of Nebula and sNebula (Figure 3a; Additional file 1: Table S2). By comparing the ACC of each HLA allele with fixed peptide length among the 7 software, we found that the median ACC of PSSMHCpan is significantly larger than other software (P <0.05, Paired T test; Figure 3b).

Table 3 Assessments (AUC values) of peptide binding affinity prediction with specific HLA alleles and peptide length by PSSMHpan, NetMHC, NetMHpan, PickPocket, Nebula, sNebula and SMM.

HLA	Length	PSSMHCpan	NetMHC*	NetMHCpan*	PickPocket*	Nebula*	sNebula*	SMM*
A*0101	9	0.96	0.98	0.98	0.94	0.82	0.97	0.97
A*0101	10	0.94	0.98	0.97	0.94	0.69	0.96	0.98
A*0201	9	0.93	0.94	0.94	0.94	0.88	0.93	0.94
A*0201	10	0.96	0.96	0.97	0.96	0.94	0.97	0.96
B*0702	9	0.95	0.97	0.97	0.96	0.81	0.95	0.97
B*0702	10	0.94	0.98	0.97	0.96	0.80	0.93	0.98
A*0202	9	0.96	0.99	0.99	0.97	0.53	0.89	0.98
A*0203	9	0.97	0.98	0.99	0.98	0.85	0.97	0.98
A*0203	10	0.95	0.98	0.98	0.95	0.53	0.96	0.97
A*6802	9	0.93	0.98	0.98	0.95	0.80	0.95	0.97
A*6802	10	0.91	0.96	0.96	0.92	0.78	0.97	0.97
B*5101	10	0.82	0.89	0.90	0.87	0.72	0.96	0.89
B*5301	9	0.93	0.98	0.98	0.96	0.55	0.88	0.98
B*5301	10	0.91	0.97	0.95	0.92	0.69	0.91	0.97
B*5401	9	0.91	0.98	0.97	0.95	0.51	0.89	0.98
B*5401	10	0.87	0.97	0.97	0.96	0.53	0.88	0.99
B*5701	9	0.98	0.99	0.99	0.98	0.94	0.99	0.99

*Training data are substantially overlapped with validation data.

Considering a one-time 10-fold cross-validation of randomly selection and non-binders construction might produce biased results, we repeated another five times of 10-fold cross-validation, and found that the standard deviations (SD) of AUCs are ≤ 0.0001, indicating no bias in the 10-fold cross-validation (Table 4).

Table 4 The AUC and SD values in 5 times 10-fold cross-validation.

Time	1	2	3	4	5	SD
PSSMHCpan	0.9405	0.9405	0.9408	0.9405	0.9406	0.0001

To evaluate pan-specific prediction of PSSMHCpan, we removed peptides in DP dataset (See Date Description) from our training data and retrained PSSMHCpan. We then predicted those peptides with PSSMHCpan, and obtained an AUC of 0.92 and an ACC of 0.86. We also predicted those peptides with NetMHCpan-3.0 and PickPocket, which gave AUC values of 0.95 and ACC values of 0.75 and 0.73, respectively. It is worth noting that the peptides that we predicted with PSSMHCpan, NetMHCpan-3.0 and PickPocket are removed from our training data, but included in the training data of NetMHCpan-3.0 and PickPocket.

In order to evaluate the pan-specificity of PSSMHCpan, we compared the allele-specific prediction with pan-specific prediction of 3,408 correctly predicted peptides. We observed a high correlation between allele-specific and pan-specific prediction (Pearson' rho=0.89, $P<0.01$; Figure 3d), suggesting that our PSSMHCpan can quantitatively predict peptide-HLA binding affinity with profound accuracy.

Mukherjee et al (2016) recently published a peptide binding affinity prediction software HLaffy that was evaluated with peptides from MHCBN and correctly detected 1179 out of 1323 binders (Table 5). To compare the performance of our PSSMHCpan with that of HLaffy, we removed the peptides in MHCBN from our training database and retrained our PSSMHCpan with the remaining peptides. Because non-binders are much less than binders in MHCBN, we only used the binders in MHCBN to evaluate and calculated the prediction accuracy by sensitivity ($Sen = \frac{TP}{\mathrm{TP+FP}}$). We found that our PSSMHCpan correctly identified 1309 out of 1323 binders (Table 5).

Table 5 Comparison of PSSMHCpan with HLaffy. The prediction of HLaffy was performed on webserver (http://proline.biochem.iisc.ernet.in/HLaffy/).

Allele	PSSMHCpan	HLaffy
HLA-A*0201	**1.00**	0.92
HLA-A*0203	**1.00**	0.93
HLA-A*0206	**1.00**	0.93
HLA-A*0301	**1.00**	0.84
HLA-A*1101	**1.00**	0.96
HLA-A*2402	**1.00**	0.77
HLA-A*3301	**1.00**	0.83
HLA-A*6801	**1.00**	0.94
HLA-A*6802	**0.95**	0.73
HLA-B*0702	**1.00**	0.88

HLA-B*3501	**0.99**	0.89
HLA-B*5301	**1.00**	0.92
HLA-B*5401	**1.00**	0.88
All	**0.99**	0.90

Evaluation of peptide binding affinity prediction with an independent dataset

Considering cross-validation might overestimate prediction accuracy, we reevaluated PSSMHCpan, NetMHC-4.0, NetMHCpan-3.0, PickPocket, Nebula, sNebula and SMM with an independent dataset that contains 273 non-duplicated experimentally verified binders from the Peptide Database of Cancer Immunity. We firstly removed 238 out of 273 binders as they are included in our training data, and then retrained the PSSMHCpan with the remaining training data. Together, we identified 268 of 273 (0.98) binders with 7 software. Of the 268 binders identified, PSSMHCpan and sNebula identified (245 and 253) substantially more binders than other 5 software did (Figure 4; Additional file 1: Table S4).

Evaluation of the peptide binding affinity prediction efficiency

As whole genome sequencing (WGS) and whole exome sequencing (WES) of cancer genome data are rapidly increasing, there is an urgent need to develop software that can quickly identify neoantigens from cancer genome data. To compare the efficiency of PSSMHCpan, NetMHC-4.0, NetMHCpan-3.0, PickPocket, Nebula, sNebula and SMM, we first calculated the predicting speed of 10-fold cross-validation on training database with 87 HLA class I alleles and found that PSSMHCpan is much faster than other six (ranging from 1.7 to 291.9 times faster; Table 6). We then used each software to independently predict binding affinity of 661,263 peptides generated from a breast tumor sample that contains 3062 somatic mutations with 6 HLA class I alleles. We found that PSSMHCpan completed the analysis in about 6 seconds. In contrast, NetMHC-4.0, took 3.61 hours, NetMHCpan-3.0 took 28.63

hours, PickPocket took 1.34 hours, sNebula took 0.35 hours and SMM took 1.49 hours to complete the analysis. Apparently, PSSMHCpan is far more efficient than other methods in detecting neoantigens from large quantity of sequencing data.

Table 6 The predicting speed (CPU time) of the seven software. The fastest ones were marked in bold.

Methods	10-fold cross-validation	Breast tumour neoantigens prediction
**PSSMHCpan**	**18.40s**	**6.34s**
**NetMHC-4.0**	1056.83s	13001.57s
**NetMHCpan-3.0**	5371.16s	103060.24s
**PickPocket**	282.83s	4839.63s
**Nebula**	146.70s	Not done
**sNebula**	31.04s	1245.88s
**SMM**	222.45s	5369.36s

CPU time was measured by second (s).

Pan-cancer neoantigens

To identify neoantigens that can be used as candidate markers to develop antitumor vaccine, we develop a neoantigen prediction pipeline to determine what types of mutated peptides in cancer cells could be brought to the cell surface by HLAs based on somatic small mutations (SSMs). In order to maximize prediction accuracy, we include PSSMHCpan, NetMHC-4.0, NetMHCpan-3.0 and PickPocket into our pipeline to detect neoantigens in TCGA tumor samples as following (Figure 5a). We first annotate missense SSMs including single nucleotide variants (SNVs), insertions and deletions (InDels) with ANNOVAR [38] to create a list of tumor-specific peptides (8-13) with an in-house script.

After HLA alleles are predicted with Seq2HLA [7], we predict neoantigens with PSSMHCpan, NetMHC-4.0, NetMHCpan-3.0 and PickPocket, respectively. Finally, we select a list of candidate neoantigens that meet the following conditions: 1) Predicting as binders (IC50<500nM) by at least 2 software and taking the median value of IC50 as final result; 2) The IC50 value of a given SNV-derived neoantigen must be smaller than that of its corresponding wild type (WT) peptide [12]. Using this pipeline, we analyzed the neoantigens across 10 cancer types from TCGA cohort.

Totally we identified candidate 117,017 neoantigens from 467 TCGA cancer samples. We calculated the number of candidate neoantigens per SSM in different types of cancer and observed that STAD, PRAD and BRCA had the highest neoantigens with 2.54, 1.52 and 1.43 per SNV, respectively (Figure 5b), whereas the highest neoantigens per InDel were 2.76, 2.59 and 2.34 in PRAD, STAD and KIRC, respectively (Figure 5c). We also compared the neoantigen loads (number of candidate neoantigens per sample) across 10 cancer types and found that STAD, COAD and BLCA tumors had the highest neoantigen loads with median values of 302, 182 and 163, while the THCA tumors had a lowest median neoantigen load of 30 (Figure 5d).

On average we identified 251 candidate neoantigens in each tumor. We then investigated whether the expression level of HLA class I would be increased in cancer cells to bind neoantigens. Indeed, by looking at the mRNA expression in 467 TCGA tumor samples and their paired normal tissues, we found that the expression of HLA class I was markedly elevated in most tumors (Figure 5e). Since the amount of candidate neoantigens differs substantially among different tumors, we examined whether the number of candidate neoantigens was correlated with HLA class I expression level in each tumor. However, we found no correlation between the number of candidate neoantigens and the HLA class I expression levels in tumors (Pearson' rho=-0.05, *P*=0.33).

Discussion

Designing antitumor vaccine requires predicting peptide-HLA binding affinity with high accuracy. In this article, we have presented a novel software PSSMHCpan that allows us to predict peptide binding affinity with a broad coverage of HLA class I alleles. By comparing our PSSMHCpan with NetMHC-4.0, NetMHCpan-3.0, PickPocket, Nebula, sNebula and SMM, we demonstrate that overall our PSSMHCpan is at least as good as the other six in predicting peptide-HLA binding affinity in terms of accuracy, and PSSMHCpan is far more efficient in detecting neoantigens from large quantity of sequencing data.

In recent years, PSSM based methods to predict peptide-HLA binding affinity were gradually replaced by machine learning based methods that are believed to have reliable accuracy and larger data prediction capability [20]. However, by comparing our PSSMHCpan with machine learning based methods NetMHC-4.0 and NetMHCpan-3.0, we show that our PSSMHCpan exhibits a higher predicting accuracy than NetMHC-4.0 and NetMHCpan-3.0 as evidenced by the independent dataset evaluation. In terms of data prediction capability, PSSMHCpan can allele-specifically and pan-specifically predict peptides that bind to 241 and 4778 HLA class I alleles, respectively. While NetMHC-4.0 and NetMHCpan-3.0 can only predict 89 and 2924 HLA class I alleles, respectively. Furthermore, the PSSMHCpan displays more than 2050 and 16255 times higher prediction efficiency as compared to NetMHC-4.0 and NetMHCpan-3.0 (Table 6).

Practically, we noticed that the size of training database appeared to directly affect the prediction accuracy. We believe that a larger training database could have improved the prediction accuracy of PSSMHCpan. For instance, the PSSMHCpan prediction accuracy ACC in predicting 9mer peptides

bind to HLA-A*0101 and HLA-B*5703 are 0.96 and 0.70. Not surprisingly, there are 813 binders for HLA-A*0101 and only 25 binders for HLA-B*5703, respectively in our training data.

It is worth noting that PSSMs with less training binders may contain more zero elements (i.e. amino acid "X" was never observed at position "Y"), which is represent as random omega in the formula of “PSSM construction” that could affect the prediction accuracy. We investigated what training binder sizes have less random omega in PSSMs, and how training binder sizes could affect prediction accuracy. There are 6,784 9mer peptides bound to HLA-A*0201 in our training database. We randomly selected 678 (10%) binders from the 6,784 9mer peptides for predicting. We then repeatedly predicted peptide binding affinity of the same 678 binders with PSSMHCpan respectively trained with increasing sizes of binders with an increment step of 10, randomly selected from the remaining 6,104 binders. We found that the prediction accuracy was increased as the training sizes increased, and the prediction accuracy reaches a plateau when the sizes of training binders are over 100 (Additional file 1: Table S5). This suggests that PSSMHCpan trained with over 100 binders would contain fewer random omegas and have stable prediction accuracy. There are less 100 training binders in 145 out of 241 PSSMs in our PSSMHCpan. In our 10-fold cross-validation, PSSMs with less than 100 training binders could have increased or decreased AUCs, with a mean value of 0.88 (ranging from 0.5 to 1). In the case of the independent dataset evaluation, 3 out of 273 binders are incorrectly predicted due to PSSMs with less than 100 training binders.

Based on the evaluation results (Figure 4), we recognized that none of the available software is perfect and that in order to maximize the peptide binding affinity prediction accuracy, it is necessary to use multiple software. We believe that in order to provide actionable neoantigens that can be used in cancer immunotherapy, it requires more efforts to validate the function and immunogenicity of the

predicted neoantigens experimentally.

In conclusion, our PSSMHCpan can predict peptide binding affinity with a broad coverage of HLA class I alleles accurately and far more efficiently compared with currently most popular peptide binding affinity prediction software. Our PSSMHCpan can not only help develop personalized antitumor vaccines, but also has great potentials in other aspects of cancer immunotherapy including designing dendritic cell (DC) vaccines, inducing DC-CTL, TCR-T, and assessing the PD-1/CTLA4 prognosis.

Availability and requirements

- Project name: PSSMHCpan
- Project home page: https://github.com/BGI2016/PSSMHCpan
- Operating system: Platform independent
- Programming language: Perl
- Other requirements: ActivePerl 5.8
- License: OSI

Availability of supporting data and materials

The supporting data from this study will be hosted in the additional files and PSSMHCpan home page.

Additional file

Additional file 1: Supplementary tables for supporting the analysis part

Table S1 is the list of HLA class I alleles and corresponding peptide length for allele-specific and pan-specific prediction. Table S2 is 10-fold cross-validation results of alleles-specific prediction of

PSSMHCpan, and the same validation on NetMHC, NetMHCpan, PickPocket, Nebula, sNebula and SMM. Table S3 is the pan-specific prediction results. Table S4 is prediction results the independent dataset. Table S5 is the Validation results of 9mer peptides bound to HLA-A*0201. The first column of "size of training database" represents the number of binder in training PSSMs.

Competing interests

The authors declare no competing financial interests.

Authors' contributions

G. L., D. L, B. L. Y. H, J. W. and H. Y. conceived of study and designed the project. G. L. and D. L. performed software development, computational analyses and prepared figures. S. Q., W. L. performed pan-cancer neoantigen analysis. G. L., B. L. and K. M. wrote the manuscript. All authors read and approved the final manuscript

Acknowledgements

We thank L. Goodman and M Dean for their constructive advices on the manuscript. We also thank Lei Ge and Yan Liang for their administrative supports. We would like to thank The Cancer Genome Atlas (TCGA), International Cancer Genome Consortium (ICGC), and Chinese Cancer Genome Consortium (CCGC) for letting us using the sequencing data. This project was supported financially with funds from the Shenzhen Science and Technology Program (JCYJ20150629114130814), the Shenzhen Municipal Government of China (KQCX20150330171652450), the National Key Research and Development Program: Precision Medical Research (2016YFC0900503, P.R. China) and application of genomic technology based early diagnosis and treatment of Qujing Xuanwei lung cancer (2016RA037, Yunan province, P.R. China)

Reference

1. Altschul SF, Gertz EM, Agarwala R et al. (2009) PSI-BLAST pseudocounts and the minimum description length principle. Nucleic acids research 37:815-824

2. Andreatta M, Nielsen M (2016) Gapped sequence alignment using artificial neural networks: application to the MHC class I system. Bioinformatics 32:511-517
3. Apweiler R, Bairoch A, Wu CH et al. (2004) UniProt: the Universal Protein knowledgebase. Nucleic acids research 32:D115-119
4. Backert L, Kohlbacher O (2015) Immunoinformatics and epitope prediction in the age of genomic medicine. Genome medicine 7:119
5. Bhasin M, Raghava GP (2007) A hybrid approach for predicting promiscuous MHC class I restricted T cell epitopes. Journal of biosciences 32:31-42
6. Bhasin M, Singh H, Raghava GP (2003) MHCBN: a comprehensive database of MHC binding and non-binding peptides. Bioinformatics 19:665-666
7. Boegel S, Lower M, Schafer M et al. (2012) HLA typing from RNA-Seq sequence reads. Genome medicine 4:102
8. Carreno BM, Magrini V, Becker-Hapak M et al. (2015) Cancer immunotherapy. A dendritic cell vaccine increases the breadth and diversity of melanoma neoantigen-specific T cells. Science 348:803-808
9. Donnes P, Kohlbacher O (2006) SVMHC: a server for prediction of MHC-binding peptides. Nucleic acids research 34:W194-197
10. Guigo R, Knudsen S, Drake N et al. (1992) Prediction of gene structure. Journal of molecular biology 226:141-157
11. Hoof I, Peters B, Sidney J et al. (2009) NetMHCpan, a method for MHC class I binding prediction beyond humans. Immunogenetics 61:1-13
12. Hundal J, Carreno BM, Petti AA et al. (2016) pVAC-Seq: A genome-guided in silico approach to identifying tumor neoantigens. Genome medicine 8:11
13. Karosiene E, Lundegaard C, Lund O et al. (2012) NetMHCcons: a consensus method for the major histocompatibility complex class I predictions. Immunogenetics 64:177-186
14. Kim Y, Sidney J, Buus S et al. (2014) Dataset size and composition impact the reliability of performance benchmarks for peptide-MHC binding predictions. BMC bioinformatics 15:241
15. Lata S, Bhasin M, Raghava GP (2007) Application of machine learning techniques in predicting MHC binders. Methods in molecular biology 409:201-215
16. Liao WW, Arthur JW (2011) Predicting peptide binding to Major Histocompatibility Complex molecules. Autoimmunity reviews 10:469-473
17. Lundegaard C, Lund O, Nielsen M (2011) Prediction of epitopes using neural network based methods. Journal of immunological methods 374:26-34
18. Luo H, Ye H, Ng H et al. (2015) Understanding and predicting binding between human leukocyte antigens (HLAs) and peptides by network analysis. BMC bioinformatics 16 Suppl 13:S9
19. Luo H, Ye H, Ng HW et al. (2016) sNebula, a network-based algorithm to predict binding between human leukocyte antigens and peptides. Scientific reports 6:32115
20. Luo H, Ye H, Ng HW et al. (2015) Machine Learning Methods for Predicting HLA-Peptide Binding Activity. Bioinformatics and biology insights 9:21-29
21. Meydan C, Otu HH, Sezerman OU (2013) Prediction of peptides binding to MHC class I and II alleles by temporal motif mining. BMC bioinformatics 14 Suppl 2:S13
22. Mukherjee S, Bhattacharyya C, Chandra N (2016) HLaffy: estimating peptide affinities for Class-1 HLA molecules by learning position-specific pair potentials. Bioinformatics

23. Nielsen M, Andreatta M (2016) NetMHCpan-3.0; improved prediction of binding to MHC class I molecules integrating information from multiple receptor and peptide length datasets. Genome medicine 8:33

24. Nielsen M, Lundegaard C, Blicher T et al. (2007) NetMHCpan, a method for quantitative predictions of peptide binding to any HLA-A and -B locus protein of known sequence. PloS one 2:e796

25. Nielsen M, Lundegaard C, Worning P et al. (2003) Reliable prediction of T-cell epitopes using neural networks with novel sequence representations. Protein science : a publication of the Protein Society 12:1007-1017

26. Noguchi H, Kato R, Hanai T et al. (2002) Hidden Markov model-based prediction of antigenic peptides that interact with MHC class II molecules. Journal of bioscience and bioengineering 94:264-270

27. Peters B, Bui HH, Frankild S et al. (2006) A community resource benchmarking predictions of peptide binding to MHC-I molecules. PLoS Comput Biol 2:e65

28. Peters B, Sette A (2005) Generating quantitative models describing the sequence specificity of biological processes with the stabilized matrix method. BMC bioinformatics 6:132

29. Robinson J, Soormally AR, Hayhurst JD et al. (2016) The IPD-IMGT/HLA Database - New developments in reporting HLA variation. Human immunology

30. Schueler-Furman O, Altuvia Y, Sette A et al. (2000) Structure-based prediction of binding peptides to MHC class I molecules: application to a broad range of MHC alleles. Protein science : a publication of the Protein Society 9:1838-1846

31. Schuler MM, Nastke MD, Stevanovikc S (2007) SYFPEITHI: database for searching and T-cell epitope prediction. Methods in molecular biology 409:75-93

32. Styczynski MP, Jensen KL, Rigoutsos I et al. (2008) BLOSUM62 miscalculations improve search performance. Nature biotechnology 26:274-275

33. Toh H, Savoie CJ, Kamikawaji N et al. (2000) Changes at the floor of the peptide-binding groove induce a strong preference for proline at position 3 of the bound peptide: molecular dynamics simulations of HLA-A*0217. Biopolymers 54:318-327

34. Trolle T, Metushi IG, Greenbaum JA et al. (2015) Automated benchmarking of peptide-MHC class I binding predictions. Bioinformatics 31:2174-2181

35. Vigneron N, Stroobant V, Van Den Eynde BJ et al. (2013) Database of T cell-defined human tumor antigens: the 2013 update. Cancer immunity 13:15

36. Vita R, Overton JA, Greenbaum JA et al. (2015) The immune epitope database (IEDB) 3.0. Nucleic acids research 43:D405-412

37. Walter S, Weinschenk T, Stenzl A et al. (2012) Multipeptide immune response to cancer vaccine IMA901 after single-dose cyclophosphamide associates with longer patient survival. Nature medicine 18:1254-1261

38. Wang K, Li M, Hakonarson H (2010) ANNOVAR: functional annotation of genetic variants from high-throughput sequencing data. Nucleic acids research 38:e164

39. Xia X (2012) Position weight matrix, gibbs sampler, and the associated significance tests in motif characterization and prediction. Scientifica 2012:917540

40. Yadav M, Jhunjhunwala S, Phung QT et al. (2014) Predicting immunogenic tumour mutations by combining mass spectrometry and exome sequencing. Nature 515:572-576

41. Zhang GL, Ansari HR, Bradley P et al. (2011) Machine learning competition in immunology -

Prediction of HLA class I binding peptides. Journal of immunological methods 374:1-4

42. Zhang H, Lund O, Nielsen M (2009) The PickPocket method for predicting binding specificities for receptors based on receptor pocket similarities: application to MHC-peptide binding. Bioinformatics 25:1293-1299

43. Zhang Q, Wang P, Kim Y et al. (2008) Immune epitope database analysis resource (IEDB-AR). Nucleic acids research 36:W513-518

FIGURE LEGENDS

Figure 1 Heat map of HLA protein sequence similarity. The larger the Z-Score, the more similar of the pair HLA protein sequences. It showed high similarity between different types of HLA alleles within the same gene locus.

Figure 2 Method of PSSMHCpan. The three mainly steps are shown in grey background.

Figure 3 Evaluation on broad HLA allelic coverage. (a) The allele-specific prediction evaluation results showed AUC and ACC value of PSSMHCpan, and also compare to NetMHC-4.0, NetMHCpan-3.0, PickPocket, Nebula, sNebula and SMM. (b) The boxplot of individual ACC of particular HLA allele with fixed peptide length. Comparison between PSSMHCpan and other six methods were performed by using paired T test. "*" denotes P<0.05 and "**" denotes P<0.01. (c) The evaluation results showed by ROC curse of PSSMHCpan in pan-specific prediction, NetMHCpan-3.0 and PickPocket. The ACC, sensitivity and specificity at cutoff of 500nM were also shown. (d) Correlation analysis of peptide-HLA binding affinity result of IC50 value in log2 between allele-specific prediction and pan-specific prediction.

Figure 4 The evaluation result of the independent dataset. We denoted IC50<500nM as binder in PSSMHCpan, NetMHC, NetMHCpan, PickPocket and SMM. In Nebula prediction, value>=1.5 as binder. In sNebula prediction, valule>=0 as binder.

Figure 5 Pan-cancer neoantigens. (a) The flow-char of neoantigen prediction pipeline. Software with

parameters using in the pipeline are shown in dashed procedure. (b) The distribution of neoantigens generated from each SNV across diverse cancers. (c) The distribution of neoantigens generated from each InDel across diverse cancers. (d) The distribution of neoantigen loads across 10 cancer types. The cancer types are sorted by median value of neoantigen loads. (e) The expression of HLA class I in tumor and corresponding normal samples.

Figure

Click here to download Figure Figure1.pdf

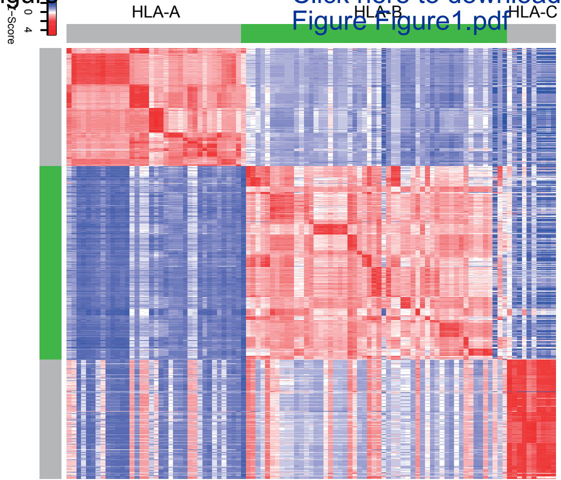

Figure Click here to download Figure Figure2.pdf

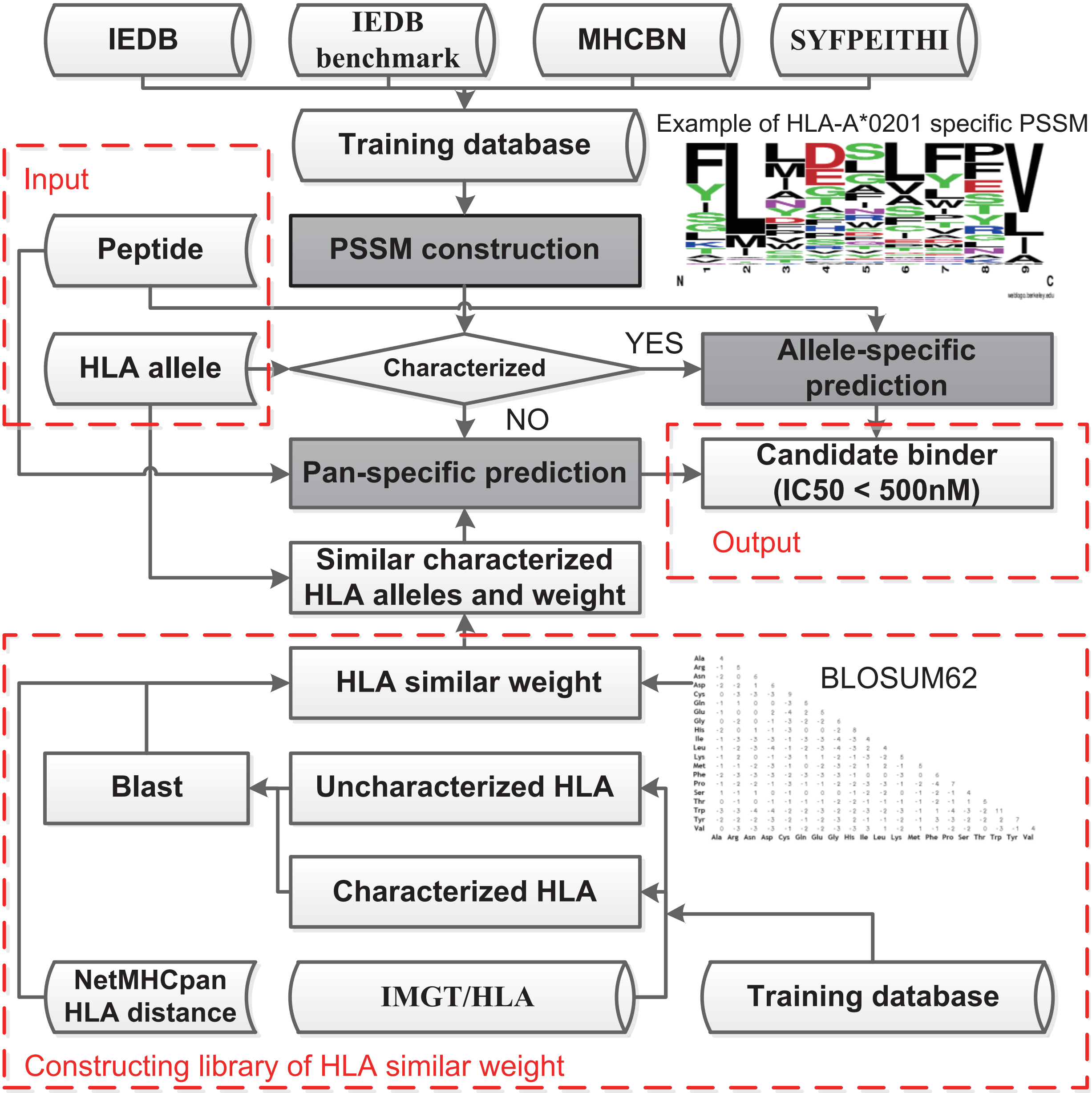

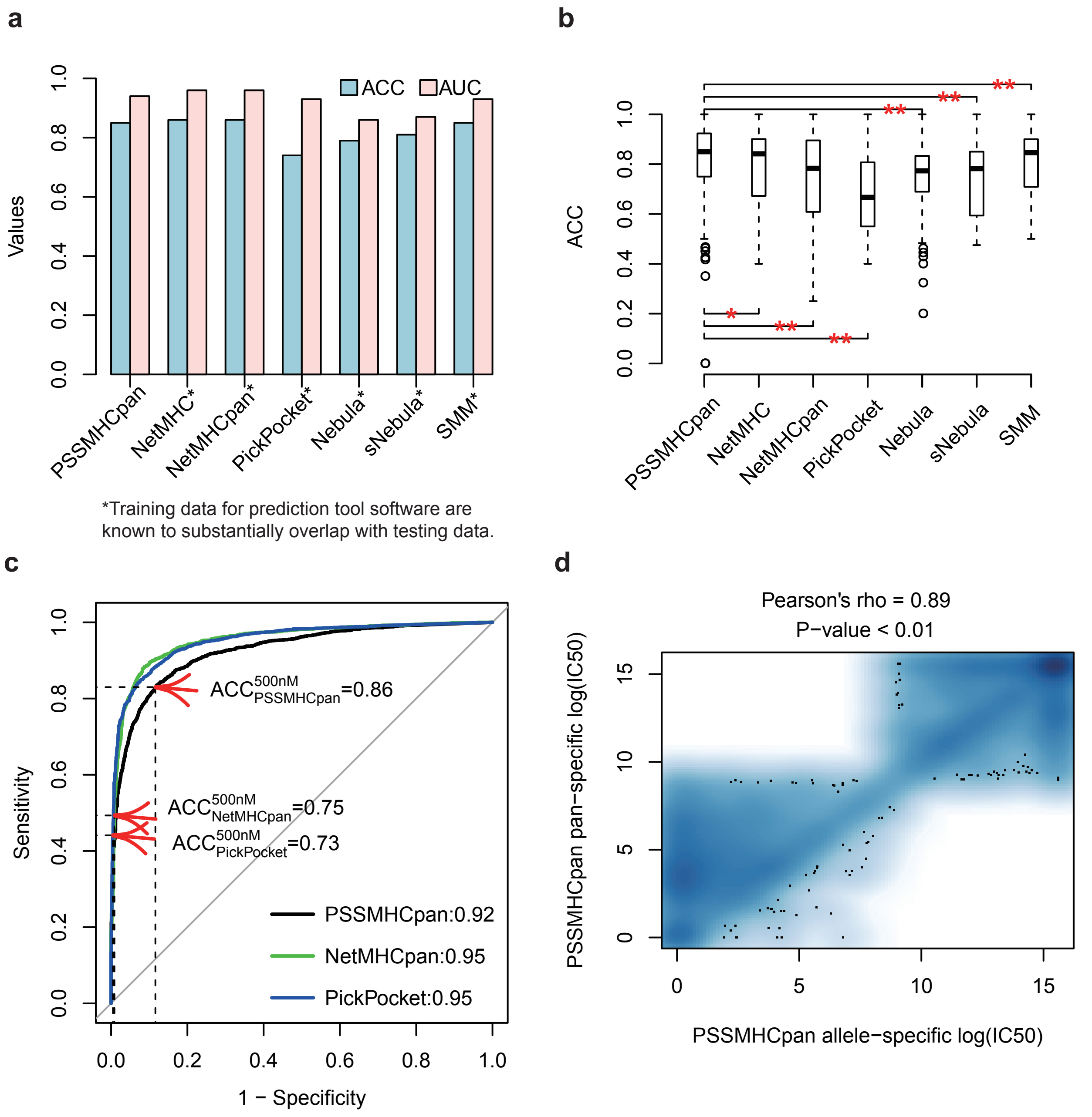
a
b
Values
ACC
AUC
PSSMHCpan
NetMHC*
NetMHCpan*
PickPocket*
Nebula*
sNebula*
SMM*
*Training data for prediction tool software are known to substantially overlap with testing data.
ACC
PSSMHCpan
NetMHC
NetMHCpan
PickPocket
Nebula
sNebula
SMM
c
d
Sensitivity
1 − Specificity
ACC 500nM PSSMHCpan =0.86
ACC 500nM NetMHCpan =0.75
ACC 500nM PickPocket =0.73
PSSMHCpan:0.92
NetMHCpan:0.95
PickPocket:0.95
Pearson's rho = 0.89
P−value < 0.01
PSSMHCpan pan−specific log(IC50)
PSSMHCpan allele−specific log(IC50)

Figure

Click here to download Figure Figure4.pdf

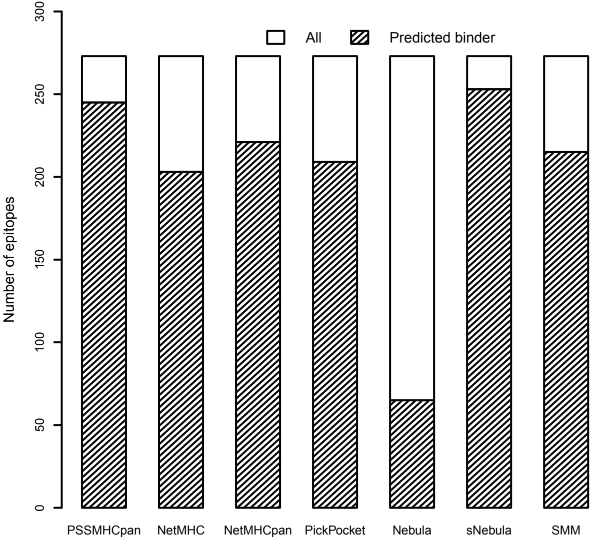

Figure Click here to download Figure Figure5.pdf

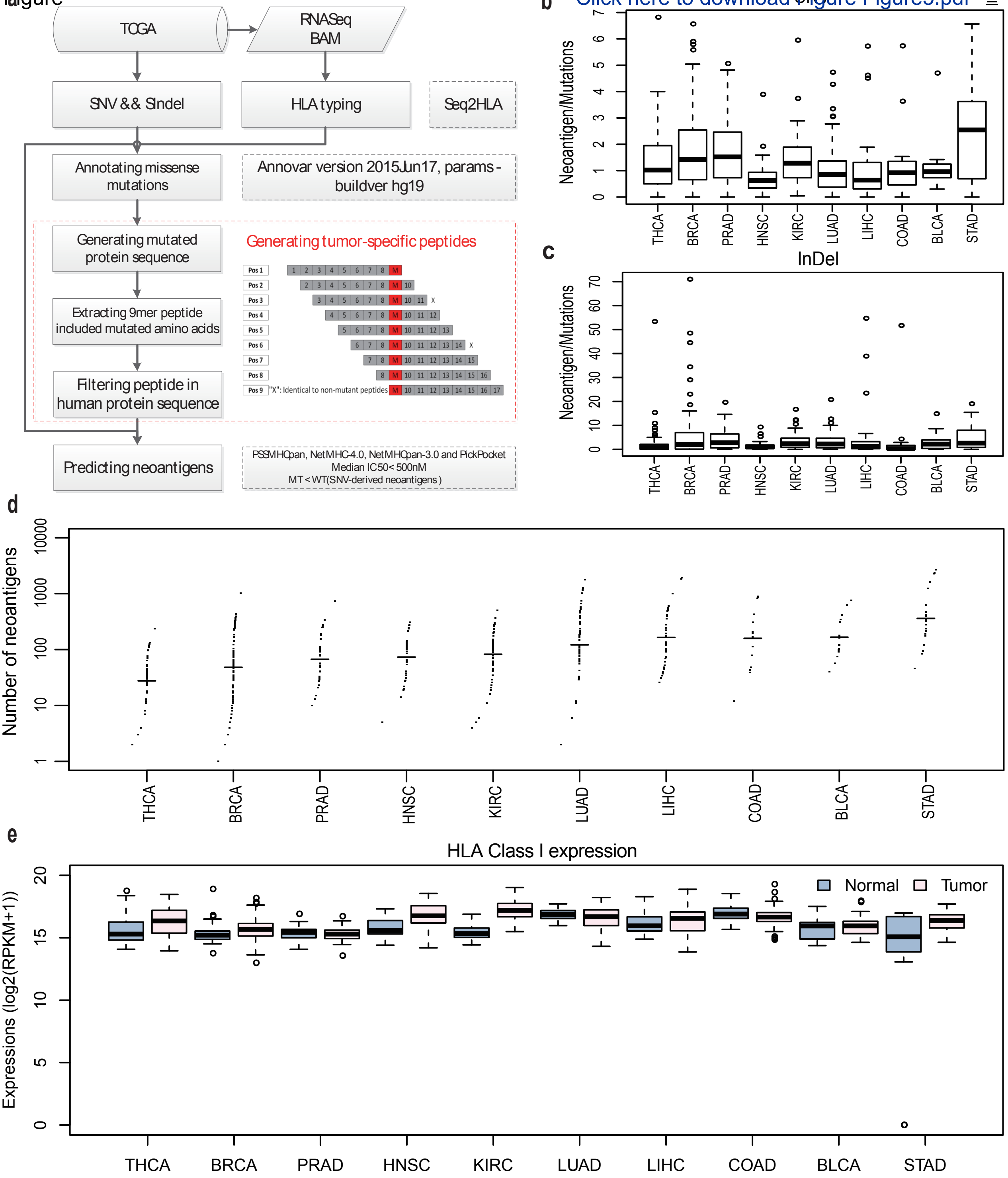

Click here to access/download
Supplementary Material
Additional file 1.xlsx
