# Supplementary material for: PSSMHCpan: a novel PSSM-based software for predicting class I peptide-HLA binding affinity
Source: Gigascience. 2017 Mar 15;6(5):1–11. doi: 10.1093/gigascience/gix017 (PMC5467046; doi:10.1093/gigascience/gix017)
Supplement: Reviewer_3_Original_Submission_(Attachement).pdf [file gix017_Reviewer_3_Original_Submission_(Attachement).pdf]

Title: PSSMHCpan: a novel PSSM based software for predicting class I peptide-HLA binding affinity

Summary and general comments:

The authors describe a PSSM based method called PSSMHCpan for prediction of HLA-peptide binding affinity. They claim that this program performs better than NetMHC-4.0, NetMHCpan-3.0 and PickPocket. The paper is neatly written but this reviewer has some concerns and recommend the paper to be revised addressing them before being considered for publication.

Major:

1. Page-1 & page-3: The authors claim that the currently available methods work well only for three class I alleles – HLA-A*02:01, A*01:01 & B*07:02. This is not correct. There are more alleles for which reliable predictions can be done using latest tools. It seems that the authors' assumption is based on the references they cited [4, 5] of which Zhang et al., 2011 is based on a machine learning competition where the data set for evaluation was limited by these 3 alleles. The other paper is published in 2003. Lot of advancement has happened in this field since 2003 and more reliable prediction algorithms have been added. This needs to be corrected or more appropriate & recent references need to be cited.
2. Page-2: "as compared to 0.85, 0.85, 0.72 in 10 cross-validations and 0.73, 0.79, 0.75 in the 28 independent dataset evaluation" – Are these values accuracy or sensitivity? What are the separate values for the two different evaluations for PSSMHCpan? The numbers mentioned in this part is not quite clear.
3. Page-3: The authors also claim "However, machine learning methods cannot accurately predict peptide binding affinity with a broad range of HLA class I allelic coverage. Further, they are inefficient in predicting peptides from a large amount of sequencing data". This reviewer does not think that this is correct. Either proper reference needs to be cited to support this claim or it needs to be proved.
4. Page-6: It is not clear what the reference is for considering "a peptide with *IC50 < 500nM* as a binder and a peptide with *IC50 < 50nM* as a strong binder". Is there any correlation between peptides with predicted IC50 < 50nM and experimentally determined as strong binders? Or is it an arbitrary consideration?
5. Page-6: Isn't the first part of the numerator & denominator same in this formula?

$$IC50_{un} = \frac{\sum_{i=1}^{S} w_i * IC50_i}{\sum_{i=1}^{S} w_i}$$

6. Page-7: "Finally, we built 241 PSSMs for allele-specific prediction of peptide binding affinity with 123 HLA class I alleles". I assume some of the PSSMs are for different peptide lengths of the same allele. This needs to be mentioned.
7. Page-11: "If a peptide binds to any 4-digital HLA allele that belong to the given 2-digital HLA allele with a predicting binding affinity IC50 less than 500nM, we considered as binder". This

reviewer does not think that it’s correct to do it this way. Binding affinity can differ widely between alleles (at 4-digit resolution level).

8. Page-12 (Table-6): PSSMHCpan took 3 times more time for cross validation data compared to breast tumor data. But the other programs took far more time for breast tumor data than cross validation data. Why is it so?
9. Page-13: 251 neoantigens have been identified on average. Is there any experimental validation for this?
10. Need proper description of supplementary data. The tables do not have legends or description. For example, What does the 2 columns in table S1 represent? Are they pairs of characterized & uncharacterized alleles? If so, why last few alleles in column 2 do not have their pairs in column 1? If not, why some alleles are duplicates in column 1?

Minor:

1. Page-1: Is PSSM “Position Specific Scoring Matrix” or “Position Score Specific Matrix”?
2. Page-1, 9: It should be mentioned “10-fold cross validation” rather than “10 cross validation”
3. Some grammar & spelling corrections are needed at several places. For example, page-12: “…PSSMHCpan are not only more accuracy but also…”, page-13: “…its corresponding wile type (WT) peptide…”
4.
