# Supplementary material for: PSSMHCpan: a novel PSSM-based software for predicting class I peptide-HLA binding affinity
Source: Gigascience. 2017 Mar 15;6(5):1–11. doi: 10.1093/gigascience/gix017 (PMC5467046; doi:10.1093/gigascience/gix017)
Supplement: Reviewer_3_Revision_1_(Attachement).pdf [file gix017_Reviewer_3_Revision_1_(Attachement).pdf]

Title: PSSMHCpan: a novel PSSM based software for predicting class I peptide-HLA binding affinity

Comments on the revised manuscript (GIGA-D-16-00055_R1):

Almost all comments were addressed satisfactorily but there three more issues that I still see which need to be addressed:

1. My comment #5 in my comments to the original 1st version of the manuscript: Page-6: Isn't the first part of the numerator & denominator same in this formula?
   I was asking this about the formula now on page 8, line 161 (in the revised manuscript):

   we qualitatively predict the binding affinity of a given peptide with uncharacterized HLA allele with an *IC50un* value which is calculated as below:

   $$IC50_{un} = \frac{\sum_{i=1}^{S} w_i * IC50_i}{\sum_{i=1}^{S} w_i}$$

   I think authors responded to this comment taking another formula into consideration.

2. The number of non-binders is not shown in Table-1 (page 9, line 188). Similarly, the no. of non-binders is not mentioned in the evaluation using independent data set (page 13, line 255 onward).

3. Page 13, line 259 onward is not clear. It says "We firstly removed 238 out of 273 binders as they are included in our training data, and then retrained the PSSMHCpan with the remaining training data. Together, we identified 268 of 273 (0.98) binders with 7 software…" I think the authors meant to say that the 238 peptides were removed from the training data set but the way it's written makes audience feel they are removed from the evaluation set. Please rephrase the sentence as needed.
