# Supplementary material for: PSSMHCpan: a novel PSSM-based software for predicting class I peptide-HLA binding affinity
Source: Gigascience. 2017 Mar 15;6(5):1–11. doi: 10.1093/gigascience/gix017 (PMC5467046; doi:10.1093/gigascience/gix017)
Supplement: Response_to_reviewer_comments_Original_Submission.pdf [file gix017_Response_to_reviewer_comments_Original_Submission.pdf]

Reviewer 1, Sebastian Maurer-Stroh:
The manuscript "PSSMHCpan: a novel PSSM based software for predicting class I peptide-HLA binding affinity" describes a straight-forward PSSM approach for allele-specific and pan-allele MHC class I peptide binder prediction. Emphasis is given to a comparative benchmark to machine-learning based methods representing the current state of the art.

It is clear that machine learning over a large parameter-space and limited number of training examples can result in seemingly good predictions overfitted to some datasets without necessarily being robust to new examples. Similarly, one has to keep in mind that also a PSSM approach uses a large parameter space of 20*peptide length 9-10 = 180-200 and is equally limited by small input sets and one should carefully consider the underlying methodology as well as independent sets and robustness tests. My detailed comments are:

1) Little information is given on how the "random non-binders" are being generated. The choice of negative examples can have implications on how realistic performance estimates and comparison with other tools turn out. 1)a) Please elaborate on "random non-binders". 1)b) Why are the authors not using experimentally verified non-binders (negative assay results from IEDB) or are they part of the described IEDB benchmark set?

Based on the reviewer's suggestion we have added experimentally verified non-binders to our non-binder validation dataset. In our revised Methods we have elaborated the "10-fold cross-validation" and "random non-binders" in lines 167-177.

2) Another test set from a cancer immune database is described as "independent". Did the authors check that the latter is not possibly partially included in the IEDB sets used for learning?

We removed 238 binders in the Peptide Database of Cancer Immunity from our training data, and used the remaining binders in the training data to retrain the PSSMHCpan and updated the validation results. We have revised this part in our manuscript in lines 255-262.

3) Alleles with few known binders are a typical problem for overfitting and robust prediction of new binders. For example, the PSSM for an allele with only 10 known 9-mer binders will have the vast number of amino acid frequencies appearing as zero (e.g. amino acid "X" was never observed at position "Y"). This means that according to their standard use of pseudocounts (formula in line 110), the score for such amino acids is determined by the random choice of parameter omega. The underlying distribution of the random function therefore has a dominant role on the score and randomly enhanced performing PSSMs could be selected during cross-validation. Certainly, the examples with worse prediction performance mentioned in the MS will relate to PSSMs with overhead of pseudocounts. The authors should investigate 1 which PSSMs are particularly affected and 2 how these perform in cross-validation or, better, truly independent sets.

We agree with the reviewer that alleles with few known binders are a problem for all types of methods for predicting peptide binding affinity. Based on the reviewer's suggestion we investigated which PSSMs are particularly affected by the random choice of parameter omega. We investigated what training binder sizes have less random omega in PSSMs, and how training binder sizes could affect prediction accuracy. We found that the prediction accuracy was increased as the training sizes increased, and the prediction accuracy reaches a plateau when the sizes of training binders are over 100. This suggests that PSSMHCpan trained with over 100 binders would contain fewer random omegas and have stable prediction accuracy. For detailed description, please see the revised manuscript in lines 334-349.

Reviewer 2, Sinu Paul:
Title: PSSMHCpan: a novel PSSM based software for predicting class I peptide-HLA binding affinity
Summary and general comments:
The authors describe a PSSM based method called PSSMHCpan for prediction of HLA-peptide binding affinity.

They claim that this program performs better than NetMHC-4.0, NetMHCpan-3.0 and PickPocket. The paper is neatly written but this reviewer has some concerns and recommend the paper to be revised addressing them before being considered for publication.

Major:

1. Page-1 & page-3: The authors claim that the currently available methods work well only for three class I alleles – HLA-A*02:01, A*01:01 & B*07:02. This is not correct. There are more alleles for which reliable predictions can be done using latest tools. It seems that the authors' assumption is based on the references they cited [4, 5] of which Zhang et al., 2011 is based on a machine learning competition where the data set for evaluation was limited by these 3 alleles. The other paper is published in 2003. Lot of advancement has happened in this field since 2003 and more reliable prediction algorithms have been added. This needs to be corrected or more appropriate & recent references need to be cited.
It is true that more reliable prediction algorithms have been developed since 2003, and there are more alleles for which reliable predictions can be done using latest tools. However, based on published literatures [22] and the MLC 2011[37], currently available tools including NetMHC, NetMHCpan and NetMHCcons can predict HLA alleles such as HLA-A*0201, A*0101 and B*0702 very well. Although current tools can predict other HLA alleles with appreciable AUCs, quite a few types of HLA alleles that are present in majority of human populations including HLA-A*0202, HLA-A*0203, HLA-A*6802, HLA-B*5101, HLA-B*5301, HLA-B*5401 and HLA-B*5701 still cannot be predicted with satisfactory accuracy using currently available methods. For example, the performance several methods in two studies [18,20] published in 2013 and 2016 showed that some of the HLA alleles (such as HLA-A*0202, HLA-A*0203, HLA-A*6802, HLA-B*5101, HLA-B*5301, HLA-B*5401 and HLA-B*5701) only achieved the average predicted AUC of no more than 0.85. We have revised the relevant part in our manuscript (lines 23-31 and 75-80).

2. Page-2: "as compared to 0.85, 0.85, 0.72 in 10 cross-validations and 0.73, 0.79, 0.75 in the 28 independent dataset evaluation" – Are these values accuracy or sensitivity? What are the separate values for the two different evaluations for PSSMHCpan? The numbers mentioned in this part is not quite clear.
0.85, 0.85, 0.72 were values of accuracy ACC, while 0.73, 0.79, 0.75 are sensitivity values. The separate values for the two different evaluations for PSSMHCpan are 0.92 and 0.87 (We have revised this part).
It is worth noting that in our revised manuscript, we based on the reviewer 1's suggestion, have added experimentally verified non-binders (IEDB) to our 10-fold cross-validation (for details please see our revised Methods, lines 167-177) and revised results. To make sure that the independent dataset evaluation we conducted is truly independent, we removed binders in the Peptide Database of Cancer Immunity from our training database, and conducted the independent dataset evaluation. We revised this part accordingly (lines 32-39).

3. Page-3: The authors also claim "However, machine learning methods cannot accurately predict peptide binding affinity with a broad range of HLA class I allelic coverage. Further, they are inefficient in predicting peptides from a large amount of sequencing data". This reviewer does not think that this is correct. Either proper reference needs to be cited to support this claim or it needs to be proved.
We agree with the reviewer's opinion. Although current tools can predict other HLA alleles with appreciable AUCs, quite a few types of HLA alleles that are present in majority of human populations including HLA-A*0202, HLA-A*0203, HLA-A*6802, HLA-B*5101, HLA-B*5301, HLA-B*5401 and HLA-B*5701 still cannot be predicted with satisfactory accuracy using currently available methods. We also explained the inefficient of machine learning method is result from its nonlinear computation complexity and will prove it by calculating the running time of different tools in the result section. Here we corrected in our MS in lines 75-82.

4. Page-6: It is not clear what the reference is for considering "a peptide with IC50 < 500nM as a binder and a peptide with IC50 < 50nM as a strong binder". Is there any correlation between peptides with predicted IC50 < 50nM and experimentally determined as strong binders? Or is it an arbitrary consideration?
"a peptide with IC50 < 500nM as a binder and a peptide with IC50 < 50nM as a strong binder" is an arbitrary consideration recommended by IEDB[39].

5. Page-6: Isn't the first part of the numerator & denominator same in this formula?
No, the numerator and denominator are different. In this formula (lines 145), the binding_score represent

the PSSM score that ranged from -1 to 1. The Max and Min is the maximum value and minimum value of binding_score. Because the binding_score can barely reach 1 or -1, we assigned Max as 0.8 and Min as -0.8 based on our experience. When the binding_score reaches maximum value, the index of this formula is 0. When the binding_score reaches minimum value, the index of this formula is 1.

6. Page-7: "Finally, we built 241 PSSMs for allele-specific prediction of peptide binding affinity with 123 HLA class I alleles". I assume some of the PSSMs are for different peptide lengths of the same allele. This needs to be mentioned.

We agree with the reviewer that some of the PSSMs are for different peptide lengths of the same allele. We have revised this part in our MS (lines 185-187).

7. Page-11: "If a peptide binds to any 4-digital HLA allele that belong to the given 2-digital HLA allele with a predicting binding affinity IC50 less than 500nM, we considered as binder". This reviewer does not think that it's correct to do it this way. Binding affinity can differ widely between alleles (at 4-digit resolution level).

We agree with the reviewer that binding affinity can differ widely between alleles in general. We have removed the sentence from our revised manuscript.

8. Page-12 (Table-6): PSSMHCpan took 3 times more time for cross validation data compared to breast tumor data. But the other programs took far more time for breast tumor data than cross validation data. Why is it so?

The input files of PSSMHCpan, NetMHC, NetMHCpan, PickPocket, Nebula, sNebula and SMM are peptide file in fasta format and HLA allele. There are 120,632 peptides that cover 87 HLA alleles in cross-validation, and 661,263 peptides that cover 6 HLA alleles in breast tumor data. Since it extremely fast to predict peptide binding affinity in PSSMHCpan, most of the running time of PSSMHCpan is spend on opening input file. The reason for which PSSMHCpan takes longer time in running cross-validation than in running breast tumor data is because that the former needs to open 87 fasta input files while the latter only needs to open 6 fasta input files.

Most of the running time of other programs is spend on predicting peptide binding affinity. That the other programs take longer time in predicting breast tumor data than in predicting cross-validation data is because breast tumor data have 661,263 peptides while cross-validation data have 120,632peptides.

9. Page-13: 251 neoantigens have been identified on average. Is there any experimental validation for this?

The average 251 neoantigens were in vitro predicted from TCGA. We did not conduct experimental validation.

10. Need proper description of supplementary data. The tables do not have legends or description. For example, What does the 2 columns in table S1 represent? Are they pairs of characterized & uncharacterized alleles? If so, why last few alleles in column 2 do not have their pairs in column 1? If not, why some alleles are duplicates in column 1?

We have added table legends to the "Additional file" in our revised manuscript.

Minor:

1. Page-1: Is PSSM "Position Specific Scoring Matrix" or "Position Score Specific Matrix"?

PSSM is "Position Specific Scoring Matrix". We have corrected this error in the revised manuscript.

2. Page-1, 9: It should be mentioned "10-fold cross validation" rather than "10 cross validation"

We have corrected this error in the revised manuscript.

3. Some grammar & spelling corrections are needed at several places. For example, page-12: "…PSSMHCpan are not only more accuracy but also…", page-13: "…its corresponding wile type (WT) peptide…"

We have corrected these errors.
