# Supplementary material for: PSSMHCpan: a novel PSSM-based software for predicting class I peptide-HLA binding affinity
Source: Gigascience. 2017 Mar 15;6(5):1–11. doi: 10.1093/gigascience/gix017 (PMC5467046; doi:10.1093/gigascience/gix017)
Supplement: Response_to_reviewer_comments_Revision_1.pdf [file gix017_Response_to_reviewer_comments_Revision_1.pdf]

Title: PSSMHCpan: a novel PSSM based software for predicting class I peptide-HLA binding affinity
Comments on the revised manuscript (GIGA-D-16-00055_R1):
Almost all comments were addressed satisfactorily but there three more issues that I still see which need to be addressed:
My comment #5 in my comments to the original 1st version of the manuscript: Page-6: Isn't the first part of the numerator & denominator same in this formula?
I was asking this about the formula now on page 8, line 161 (in the revised manuscript):

we qualitatively predict the binding affinity of a given peptide with uncharacterized HLA allele with an IC50un value which is calculated as below:

I think authors responded to this comment taking another formula into consideration.
Answer:
We assume that the reviewer might think that ∑wi has the same thing in numerator and denominator. It is not. The part of ∑wi in numerator could not be separated from IC50i. In order to make this formula easy to understand, we revise it as in line 161.

The number of non-binders is not shown in Table-1 (page 9, line 188). Similarly, the no. of non-binders is not mentioned in the evaluation using independent data set (page 13, line 255 onward).
Answer:
We only use experientially verified binders to train our PSSMHCpan. The summary of the training data is shown in Table-1 which did not show the number of non-binders. The number of binders and non-binders are the same in our 10-fold cross-validation (See Table-S2 and Table-S3).
In the independent data set evaluation, we use the Peptide Database of Cancer Immunity which only consists of experientially verified binders. We did not use non-binders in the independent data set evaluation.

Page 13, line 259 onward is not clear. It says "We firstly removed 238 out of 273 binders as they are included in our training data, and then retrained the PSSMHCpan with the remaining training data. Together, we identified 268 of 273 (0.98) binders with 7 software…" I think the authors meant to say that the 238 peptides were removed from the training data set but the way it's written makes audience feel they are removed from the evaluation set. Please rephrase the sentence as needed.
Answer:
We rephrase the sentence as follows in lines 268-270: "Of the 273 binders, 238 are included in our training data. In order to perform independent evaluation, we firstly removed the 238 binders from our training data, and then retrained the PSSMHCpan with the remaining training data."
