# Supplementary material for: PSSMHCpan: a novel PSSM-based software for predicting class I peptide-HLA binding affinity
Source: Gigascience. 2017 Mar 15;6(5):1–11. doi: 10.1093/gigascience/gix017 (PMC5467046; doi:10.1093/gigascience/gix017)
Supplement: Reviewer_2_Report_(Original_Submission).pdf [file gix017_Reviewer_2_Report_(Original_Submission).pdf]

**viewer Report**

**Title:** "PSSMHCpan: a novel PSSM based software for predicting class I peptide-HLA binding affinity"

**Version:** Original Submission **Date:** 8/15/2016

**Reviewer name:** Sebastian Maurer-Stroh

**Reviewer Comments to Author:**

The manuscript "PSSMHCpan: a novel PSSM based software for predicting class I peptide-HLA binding affinity" describes a straight-forward PSSM approach for allele-specific and pan-allele MHC class I peptide binder prediction. Emphasis is given to a comparative benchmark to machine-learning based methods representing the current state of the art.

It is clear that machine learning over a large parameter-space and limited number of training examples can result in seemingly good predictions overfitted to some datasets without necessarily being robust to new examples. Similarly, one has to keep in mind that also a PSSM approach uses a large parameter space of 20*peptide length 9-10 = 180-200 and is equally limited by small input sets and one should carefully consider the underlying methodology as well as independent sets and robustness tests. My detailed comments are:

1) Little information is given on how the "random non-binders" are being generated. The choice of negative examples can have implications on how realistic performance estimates and comparison with other tools turn out. 1)a) Please elaborate on "random non-binders". 1)b) Why are the authors not using experimentally verified non-binders (negative assay results from IEDB) or are they part of the described IEDB benchmark set?

2) Another test set from a cancer immune database is described as "independent". Did the authors check that the latter is not possibly partially included in the IEDB sets used for learning?

3) Alleles with few known binders are a typical problem for overfitting and robust prediction of new binders. For example, the PSSM for an allele with only 10 known 9-mer binders will have the vast number of amino acid frequencies appearing as zero (e.g. amino acid "X" was never observed at position "Y"). This means that according to their standard use of pseudocounts (formula in line 110), the score for such amino acids is determined by the random choice of parameter omega. The underlying distribution of the random function therefore has a dominant role on the score and randomly enhanced performing PSSMs could be selected during cross-validation. Certainly, the examples with worse prediction performance mentioned in the MS will relate to PSSMs with overhead of pseudocounts. The authors should investigate which PSSMs are particularly affected and how these perform in cross-validation or, better, truly independent sets.

**Methods**

Are the methods appropriate to the aims of the study, are they well described, and are necessary controls included? No

**Conclusions**

Are the conclusions adequately supported by the data shown? No

**Reporting Standards**

Does the manuscript adhere to the journal's guidelines on minimum standards of reporting? No

**Statistics**

Are you able to assess all statistics in the manuscript, including the appropriateness of statistical tests used? Yes, and I have assessed the statistics in my report.

**Quality of Written English**

Please indicate the quality of language in the manuscript: Needs some language corrections before being published

**Declaration of Competing Interests**

Please complete a declaration of competing interests, considering the following questions:

- Have you in the past five years received reimbursements, fees, funding, or salary from an organisation that may in any way gain or lose financially from the publication of this manuscript, either now or in the future?
- Do you hold any stocks or shares in an organisation that may in any way gain or lose financially from the publication of this manuscript, either now or in the future?
- Do you hold or are you currently applying for any patents relating to the content of the manuscript?
- Have you received reimbursements, fees, funding, or salary from an organization that holds or has applied for patents relating to the content of the manuscript?
- Do you have any other financial competing interests?
- Do you have any non-financial competing interests in relation to this paper?

If you can answer no to all of the above, write 'I declare that I have no competing interests' below. If your reply is yes to any, please give details below.

I declare that I have no competing interests

I agree to the open peer review policy of the journal. I understand that my name will be included on my report to the authors and, if the manuscript is accepted for publication, my named report including any attachments I upload will be posted on the website along with the authors' responses. I agree for my report to be made available under an Open Access Creative Commons CC-BY license (http://creativecommons.org/licenses/by/4.0/). I understand that any comments which I do not wish to be included in my named report can be included as confidential comments to the editors, which will not be published.

I agree to the open peer review policy of the journal

To further support our reviewers, we have joined with Publons, where you can gain additional credit to further highlight your hard work (see: https://publons.com/journal/530/gigascience). On publication of this paper, your review will be automatically added to Publons, you can then choose whether or not to claim your Publons credit. I understand this statement.

Yes
