# Supplementary material for: PSSMHCpan: a novel PSSM-based software for predicting class I peptide-HLA binding affinity
Source: Gigascience. 2017 Mar 15;6(5):1–11. doi: 10.1093/gigascience/gix017 (PMC5467046; doi:10.1093/gigascience/gix017)
Supplement: Reviewer_3_Report_(Revision_1).pdf [file gix017_Reviewer_3_Report_(Revision_1).pdf]

Reviewer Report

Title: "PSSMHCpan: a novel PSSM based software for predicting class I peptide-HLA binding affinity"

Version: Revision 1 **Date:** 1/13/2017

Reviewer name: Sinu Paul

Reviewer Comments to Author:

Almost all comments were addressed satisfactorily but there three more issues that I still see which need to be addressed:

1) My comment #5 in my comments to the original 1st version of the manuscript: Page-6: Isn't the first part of the numerator & denominator same in this formula?

I was asking this about the formula now on page 8, line 161 (in the revised manuscript pdf) ("we qualitatively predict the binding affinity of a given peptide with uncharacterized HLA allele with an IC50un value which is calculated as below:")

I think authors responded to this comment taking another formula into consideration.

2) The number of non-binders is not shown in Table-1 (page 9, line 188). Similarly, the no. of non-binders is not mentioned in the evaluation using independent data set (page 13, line 255 onward).

3) Page 13, line 259 onward is not clear. It says "We firstly removed 238 out of 273 binders as they are included in our training data, and then retrained the PSSMHCpan with the remaining training data. Together, we identified 268 of 273 (0.98) binders with 7 software..." I think the authors meant to say that the 238 peptides were removed from the training data set but the way it's written makes audience feel they are removed from the evaluation set. Please rephrase the sentence as needed.

Methods

Are the methods appropriate to the aims of the study, are they well described, and are necessary controls included? Yes

Conclusions

Are the conclusions adequately supported by the data shown? Yes

Reporting Standards

Does the manuscript adhere to the journal's guidelines on minimum standards of reporting? Yes

Statistics

Are you able to assess all statistics in the manuscript, including the appropriateness of statistical tests used? Yes, and I have assessed the statistics in my report.

Quality of Written English

Please indicate the quality of language in the manuscript: Acceptable

Declaration of Competing Interests

Please complete a declaration of competing interests, considering the following questions:

- Have you in the past five years received reimbursements, fees, funding, or salary from an organisation that may in any way gain or lose financially from the publication of this manuscript, either now or in the future?
- Do you hold any stocks or shares in an organisation that may in any way gain or lose financially from the publication of this manuscript, either now or in the future?
- Do you hold or are you currently applying for any patents relating to the content of the manuscript?
- Have you received reimbursements, fees, funding, or salary from an organization that holds or has applied for patents relating to the content of the manuscript?
- Do you have any other financial competing interests?
- Do you have any non-financial competing interests in relation to this paper?

If you can answer no to all of the above, write 'I declare that I have no competing interests' below. If your reply is yes to any, please give details below.

I declare that I have no competing interests

I agree to the open peer review policy of the journal. I understand that my name will be included on my report to the authors and, if the manuscript is accepted for publication, my named report including any attachments I upload will be posted on the website along with the authors' responses. I agree for my report to be made available under an Open Access Creative Commons CC-BY license (http://creativecommons.org/licenses/by/4.0/). I understand that any comments which I do not wish to be included in my named report can be included as confidential comments to the editors, which will not be published.

I agree to the open peer review policy of the journal

To further support our reviewers, we have joined with Publons, where you can gain additional credit to further highlight your hard work (see: https://publons.com/journal/530/gigascience). On publication of this paper, your review will be automatically added to Publons, you can then choose whether or not to claim your Publons credit. I understand this statement.

Yes
